# The genome of a sea spider corroborates a shared Hox cluster motif in arthropods with a reduced posterior tagma

**DOI:** 10.1186/s12915-025-02276-x

**Published:** 2025-07-02

**Authors:** Nikolaos Papadopoulos, Siddharth S. Kulkarni, Christian Baranyi, Bastian Fromm, Emily V. W. Setton, Prashant P. Sharma, Andreas Wanninger, Georg Brenneis

**Affiliations:** 1 Integrative Zoology Unit, Department of Evolutionary Biology, University of Vienna, Vienna, Austria; 2 CSIR-Centre for Cellular and Molecular Biology, Hyderabad, India; 3 The Arctic University Museum of Norway, UiT - The Arctic University of Norway, Tromsø, Norway; 4 Department of Integrative Biology and Zoological Museum, University of Wisconsin-Madison, Madison, WI USA; 5 The Whitney Laboratory for Marine Bioscience, Department of Biology, University of Florida, St. Augustine, FL USA

**Keywords:** Chelicerata, Pycnogonida, *Pycnogonum litorale*, Evolution, Body plan, *Abdominal-A*, Whole-genome duplication

## Abstract

**Background:**

Chelicerate evolution is contentiously debated, with recent studies challenging traditional phylogenetic hypotheses and scenarios of major evolutionary events, like terrestrialization. Sea spiders (Pycnogonida) represent the uncontested marine sister group of all other chelicerates, featuring a—likely plesiomorphic—indirect development. Accordingly, pycnogonids hold the potential to provide crucial insight into the evolution of chelicerate genomes and body patterning. Due to the lack of high-quality genomic and transcriptomic resources, however, this potential remains largely unexplored.

**Results:**

We employ long-read sequencing and proximity ligation data to assemble the first near chromosome-level sea spider genome for *Pycnogonum litorale*, complemented by comprehensive transcriptomic resources. The assembly has a size of 471 Mb in 57 pseudochromosomes, a repeat content of 61.05%, 15,372 predicted protein-coding genes, and robust completeness scores (95.8% BUSCO Arthropoda score, 95.7% of conserved microRNA families). Genome-scale self-synteny and homeobox gene cluster analysis show no evidence of a whole-genome duplication (WGD). We identify a single, intact Hox cluster lacking *Abdominal-A* (*abdA/Hox9*), corroborated by the absence of an *abdA* ortholog in the novel transcriptomic resources.

**Conclusions:**

Our high-quality genomic and transcriptomic resources establish *P. litorale* as a key research organism for modern studies on chelicerate genome evolution, development, and phylogeny. The lack of WGD signature in *P. litorale* further strengthens the inference that WGDs are derived traits in the chelicerate tree. The combination of *abdA* loss with the reduction of the posterior tagma emerges as a common theme in arthropod evolution, as it is shared with other, distantly related arthropod taxa with a vestigial opisthosoma/abdomen.

**Supplementary Information:**

The online version contains supplementary material available at 10.1186/s12915-025-02276-x.

## Background

Chelicerata represents an extremely diverse arthropod lineage boasting more than 120,000 extant species [1]. They inhabit a wide range of habitats and have adopted highly divergent life strategies, as impressively evidenced by extant terrestrial arachnid taxa (such as spiders, scorpions, harvestmen, mites, and ticks). In contrast to most of their chelicerate kin, horseshoe crabs (Xiphosura) and sea spiders (Pycnogonida), plus selected mite taxa, are the only extant groups that inhabit the oceans [2, 3].

Paramount to the chelicerate radiation has been the evolutionary plasticity of their body plan. One of its widely conserved hallmarks is the presence of two tagmata: the prosoma and the opisthosoma [4]. The anterior prosoma comprises the ocular region and typically (but not always) six segments bearing the eponymous raptorial chelicera, followed by the pedipalp and four pairs of legs, all of which have been subject to considerable transformations in different lineages. By contrast, the posterior opisthosoma displays far greater evolutionary plasticity across chelicerates, not only in terms of segment number (up to 12 plus the asegmental terminus, the telson), but also with regard to the presence and function of diverse appendage derivatives (e.g., book gills, book lungs, or spinnerets) [4, 5].

**Fig. 1:**
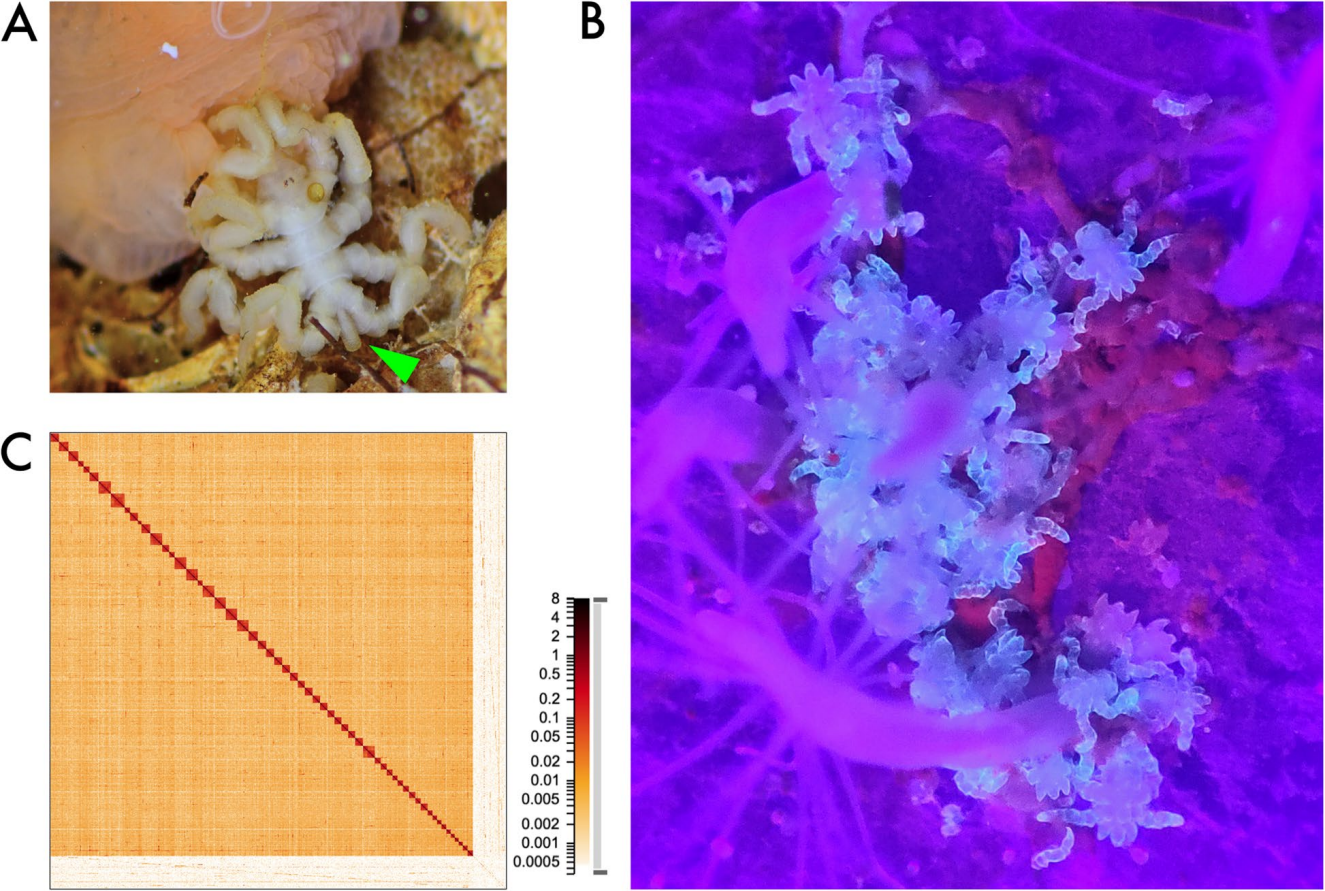
**A** Adult *Pycnogonum litorale* specimen (approx. 1 cm body length without proboscis), feeding on the sea anemone *Metridium senile*. The green arrowhead points to the vestigial opisthosoma/anal tubercle. **B** Post-embryonic instars V of *P. litorale* (fluorescing in light blue under UV light exposure; approx. 0.5–0.6 mm in size), feeding on the hydrozoan *Clava multicornis*. **C** Chromatin contact map, generated from Omni-C data at 1 kb resolution, showing the 57 pseudochromosomes of the haploid genome (dark squares) and the unscaffolded contigs (bottom right region). Color denotes the number of contacts found in each region

The body organization of sea spiders is a unique variation on the chelicerate theme, characterized by several lineage-specific traits. The prosoma carries a prominent suctorial apparatus (proboscis), a heavily modified first leg (oviger) used for egg-carrying and grooming, and at least four pairs of true walking legs [6]. However, three distantly related pycnogonid taxa even possess five or six leg pairs [7–9], which showcases a variability in the segmental composition of the prosoma that is uncharacteristic for the other chelicerate taxa. On the other hand, the opisthosoma of pycnogonids is dramatically reduced and represents only a small posterior protrusion (anal tubercle or “abdomen”) (Fig. 1A). Notably, it remains unclear to what extent vestigial opisthosomal segments contribute to the anal tubercle (e.g., [10]).

Notwithstanding their peculiar adult body organization, pycnogonids are the only extant chelicerates that display pronounced indirect development: the primary larva that hatches from the egg features only three appendage-bearing segments and subsequently undergoes anamorphic development with sequential body segment addition at the posterior pole. This trait may not only resemble the ancestral condition of chelicerates but also of arthropods in general [11–13]. Accordingly, the study of sea spider development holds the potential to crucially inform debates on the evolutionary trajectories of chelicerate body patterning [14, 15]. This is further underscored by their position in the chelicerate tree of life. After a long phylogenetic odyssey [16], pycnogonids are now robustly established as sister group of all other taxa (= Euchelicerata), rendering them one of the few stable anchors in the historically contentious and still controversial higher-order phylogeny of chelicerates [17–22].

This stable pycnogonid-euchelicerate sister group relationship also highlights a key role for sea spiders in interpreting the evolution of chelicerate genomes. For instance, the recently proposed hypothesis that whole-genome duplication (WGD) events in arachnopulmonates (spiders, scorpions and kin) and in xiphosurans [23–26] represent derived states within chelicerates (e.g., [25]) is based on the lack of extensive duplications in so-called apulmonate taxa (harvestmen, ticks, mites) [27–30]. However, with euchelicerate interrelationships still in flux and no high-quality genomic resources for pycnogonids, polarization of WGD events and the reconstruction of the ancestral chelicerate condition remain challenging.

Similarly, the current availability of but a single scaffold-level draft genome for *Nymphon striatum* [31] and a handful of bulk transcriptomes from limited developmental stages [7] has prevented inclusion of pycnogonids in macroevolutionary comparative studies. This includes studies on the composition of the Hox gene cluster, one of the best-known and most extensively discussed syntenic motifs of metazoan genomes (e.g., [32]). In euchelicerates, Hox gene cluster duplications provided the first hints for WGD events (e.g., [24]). Functionally, Hox genes play a crucial role in the specification of segment identity along the anterior–posterior body axis of arthropods [33]. This renders them crucial targets in the study of the genetic underpinnings of some of the unique features of the pycnogonid body plan.

The sea spider species *Pycnogonum litorale* (Strøm, 1762) from the north Atlantic is an emerging laboratory organism that allows us to bridge this knowledge gap. It can be kept in laboratory cultures (Fig. 1A, B), is long-lived, and displays year-round reproduction with a few thousand eggs per mating [34, 35]. In addition to these favorable characteristics, a solid body of morphological data on ontogeny and adult morphology are available for this species (e.g., [36–39]). As a result, it has contributed to a number of developmental genetic investigations, both in standalone works as well as comparative studies [10, 40–42]. To complement this morphological foundation and overcome the limitations of the molecular resources available for pycnogonids, we present here the first genome of *P. litorale* together with novel transcriptomes for a closely spaced series of embryonic stages and post-embryonic instars of its life cycle.

## Results

### Genome sequencing and assembly

Specimen H1 was sequenced to 30.5 M reads (56.4 Gb) with the Oxford Nanopore Technologies (ONT) PromethION platform (N50: 5.3 kb, average read length: 1.85 kb). The M1 specimen was sequenced with the Pacific Biosciences (PacBio) HiFi platform to 1.7 M reads (10.1 Gb). K-mer spectrum analysis on the GenomeScope [43] and GenomeScope2 [44] webservers predicted medium levels of heterozygosity (approx. 0.68%; see Methods for caveats). The predicted genome size was between 190 and 530 Mb (Additional File 1: Fig. S1), a range below the only other currently available pycnogonid draft genome of *Nymphon striatum* at approx. 732 Mb [31]. The low k-mer coverage of the PacBio reads (8–17x, see Additional File 1: Fig. S1A, C) discouraged us from using them for de novo genome assembly. Specimen M2 was sequenced to 79.4 M reads (24 Gb) using the Omni-C protocol and the Illumina platform. For an overview of sequencing data, see Additional File 2: Table 1.

To complement the genomic data, we generated 1.24B Illumina short transcriptomic reads (186.7 Gbp) from 15 closely sampled developmental time points, spanning from the zygote to the sub-adult stage (Additional File 2: Table 1; refer to [37–39] for names and descriptions of the developmental stages). We also generated 1.5 M full-length mRNA reads (3.43 Gb) using the PacBio Iso-seq technology.

We tried a variety of de novo genome assemblers using both the ONT and HiFi data. A comprehensive table of assembly results can be found in the GitLab repository [45] under 01-assemble. The most promising assembly in terms of contiguity and completeness was produced with the ONT data using Flye. After scaffolding, manual curation of the Omni-C map, merging of smaller scaffolds by their Omni-C score, and scaffold decontamination (see Methods), the draft genome had a total size of 471.6 Mb and an N50 of approx. 8 Mb, with almost 93% of the sequences contained in 57 pseudochromosomes (in silico assembled pseudomolecules not verified experimentally) (Fig. 1C). To assess completeness, we used the arthropod set of the Benchmarking Universal Single-Copy Ortholog database (BUSCO, [46]), reaching a completeness of 95.8%. The completeness and contiguity progress from initial assembly, through scaffolding, manual curation, and decontamination is documented in Additional File 3: Table 2. The majority of genomic and transcriptomic raw reads mapped back to the draft assembly (ONT: 76.23%, PacBio: 84.38%, short RNA average: 87.75%, Iso-seq: 99.67%), indicating that the assembly captures the genetic variability of the laboratory culture and the wildtype specimen equally well.

### Genome annotation

#### Repeat annotation and analysis

**Fig. 2:**
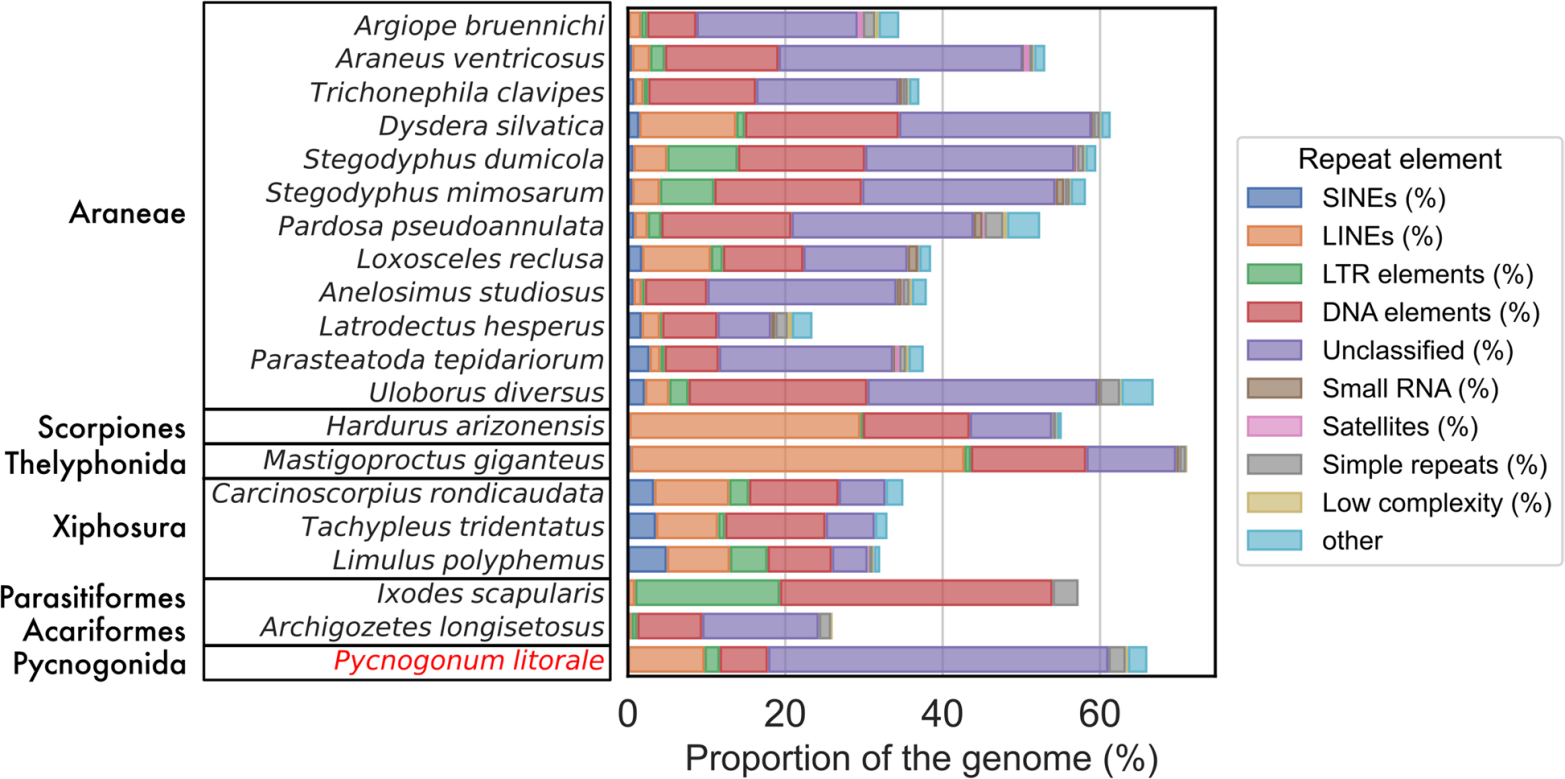
Breakdown of repeat content for various chelicerate genomes, including *Pycnogonum litorale* (highlighted in red font). Araneae as reported in [47]; *H. arizonensis* as reported in [48]; *M. giganteus* as reported in [49]; Xiphosura as reported in [50]; *I. scapularis* as reported in [51]; *A. longisetosus* as reported in [27]. For underlying data, see Additional File 17: Table 8

**Fig. 3:**
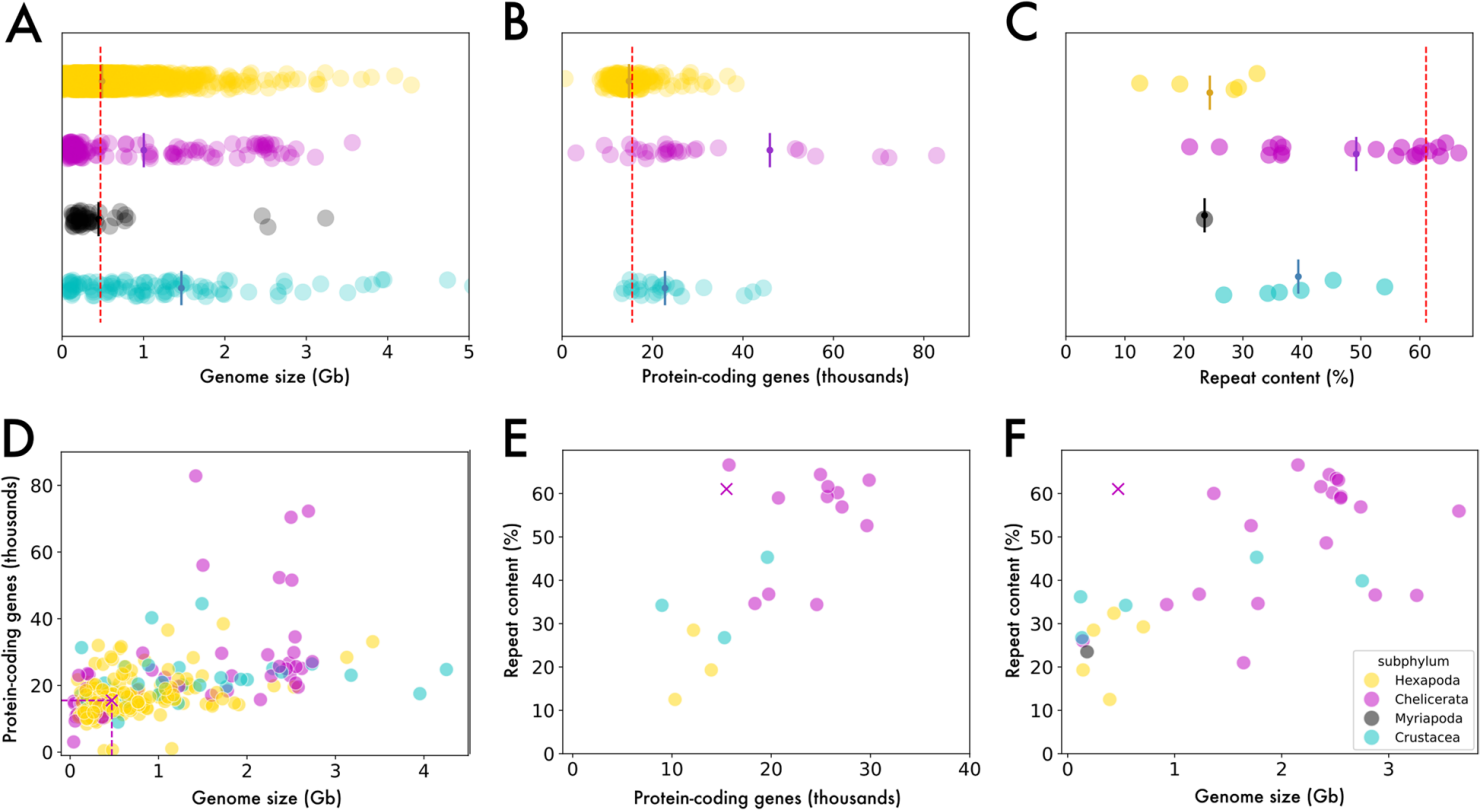
The *Pycnogonum litorale* genome in the broader arthropod genomic context. Each point represents one genome, with Hexapoda shown in yellow, chelicerates in magenta, myriapods in gray, and crustaceans in cyan. **A–C** Strip plots of the distributions of **A** genome size, **B** predicted protein-coding genes, and **C** repeat content. The mean of each distribution is indicated by a bisected circle. The values for the *P. litorale* genome are indicated by a dashed red line. **D–F** Scatter plots showing the relationship of **D** protein-coding gene number to genome size, **E** repeat content to protein-coding genes, and **F** repeat content to genome size. The values of the *P. litorale* genome are indicated by a cross. Data from NCBI (Additional File 18: Table 9, Additional File 19: Table 10, and Additional File 20: Table 11))

Using RepeatModeler and RepeatMasker, a total of 61.05% of the assembly was identified as repetitive elements, with 11.65% being classified as retroelements (mostly long interspersed nuclear elements, at 9.75%), 6.14% classified as DNA transposons, and 43.26% remaining unclassified (Fig. 2). As this level of repeat content is unusual for the modest genome size (see Fig. 3F), it prompted a deeper analysis. We first sought to confirm that the high amount of repetitive sequences was not an artifact of the genome assembly. To this end, we mapped the raw ONT and PacBio reads onto the repeat families predicted by RepeatModeler. We found close to 97 million matches on 18.5 million ONT reads (total: 30.5 million, for a 60.41% mapping rate), indicating that each read contained on average more than 5 repeats. The results were similar for the PacBio data, with 11 million matches on 1.37 million HiFi reads (total: 1.72 million, for an 80.1% mapping rate), indicating that each read contained on average 8 repeats. The high abundance of repeated sequences on the raw reads of two different sequencing technologies and specimens originating from different natural populations leads us to conclude that the annotated repeat content is likely not a prediction artifact. The repeat analysis results are available online in the GitLab repository [45] under 04-contam/.

Next we investigated the unclassified repeats, as they made up the majority of the repeat content of *P. litorale.* Manual inspection of some unclassified repeat sequences retrieved low-confidence database hits (nucleotide BLAST against the NCBI nr database) from sea anemones, including *Metridium senile*, which is the prey of juveniles, subadults, and adults of *P. litorale* in our laboratory culture. To exclude contamination as a possible explanation, we identified 10,170 raw ONT reads (0.03% of total reads) that mapped with high confidence (Q > 30) to both the *P. litorale* and *M. senile* genomes. Of these, more than half (4593) are highly repetitive (> 50% of read length) and another 14% (1446) are mildly repetitive (> 25% of read length). Furthermore, many of the affected *P. litorale* contigs are themselves highly repetitive, with one contig in particular containing 2197 of the suspicious reads. Accordingly, the putative contaminants from *M. senile* are a vanishingly small number of highly repetitive reads on highly repetitive contigs (see also relevant notebooks in the GitLab repository under 04-contam/). We consider the danger of contamination or misassembly to be rather low.

#### Gene prediction

Using the short-read transcriptome data for protein-coding gene prediction with BRAKER [52] yielded 11,451 gene models. However, suboptimal BUSCO scores (lower completeness, higher duplication rate) of the gene models (“protein” mode) compared to the entire genome (“genome” mode) prompted us to investigate the predictions. We cross-referenced this with the loci of the 8904 clusters produced by the Iso-seq processing pipeline (isoform collapsing, see Material and Methods) and were able to identify multiple cases where BRAKER models of distinct transcript clusters were erroneously merged, suggesting that BRAKER did not detect the intergenic boundaries correctly. We also noticed a number of loci with RNA-seq peaks in characteristic exon–intron patterns without gene models, suggesting that, in these loci, the BRAKER annotation had been too conservative. We resolved these cases by treating Iso-seq transcript clusters as genes and ignoring BRAKER predictions that overlapped (same strand, any sequence overlap) with Iso-seq “genes.” This left 3596 BRAKER gene models to complement the Iso-seq clusters, leading to a total of 12,500 gene models. We then filtered the short-read transcriptome data to exclude reads that mapped to loci with gene models and ran BRAKER again. This produced 2223 gene models that did not overlap with the first round of annotation. We verified by manual inspection that many previously unidentified loci were now covered by gene models. Finally, we used the de novo assembled transcriptomes from deeply sequenced developmental time points to suggest another 754 putative gene loci, for a total of 15,497 gene models. Of these, we manually merged three models that were erroneously split, and merged further duplicated loci, leading to a final total of 15,372 genes. The vast majority of genes (95.7%) are on the 57 pseudochromosomes, with only 670 gene models on smaller scaffolds. The completeness score for BUSCO Arthropoda remains stable (“genome” mode on the raw sequence of the draft genome: 95.8%, “protein” mode on the predicted proteome: 95.8%). A total of 10,017/15,372 (approx. 65%) of the gene models were assigned an orthogroup by EggNOG-mapper, meaning they mapped to known gene families. Finally, prediction of tRNA genes resulted in a total of 4189 genes, of which 965 were not overlapping with protein-coding genes.

#### MicroRNA content

Of the 47 conserved microRNA families predating the evolutionary origin of Chelicerata (1 Eumetazoa, 31 Bilateria, 11 Protostomia, 1 Ecdysozoa and 3 Arthropoda; see [53]), we found 42 in the high-confidence predictions, indicating a very good performance of the Covariance models in MirMachine. Missing families included the bilaterian-specific Mir-242 and Mir-76 (with Mir-242 being a known loss in all Ecdysozoa), the protostome-specific Mir-1993 and Mir-67, and Iab-4. Manual investigation of the low-confidence candidates for these families identified predictions for Mir-76, Mir-1993, and Mir-67 with conserved seed sequences, likely representing true microRNAs. Hence, with 45 out of the 47 clearly expected families (95.7%), the genome assembly reaches a high completeness score for conserved microRNAs, serving as an additional indicator of its high quality. By contrast, out of the three microRNA families hitherto classified as being chelicerate-specific (Mir-3931, Mir-5305, and Mir-5735), none were recovered in the high-confidence predictions. However, a low-confidence prediction with a well-conserved seed sequence likely resembles a true positive for the Mir-3931 family. Of the predicted microRNA families, 11 had more than one copy, leading to a total of 70 predicted microRNA genes (Additional File 4: Table 3).

The intermediate and final microRNA annotations are available on Zenodo in the generic feature format (GFF) (https://explore.openaire.eu/search/dataset?pid=10.5281%2Fzenodo.14362378), including functional predictions; the assembly, including gene models but lacking functional annotation, is also deposited on the ENA server under accession ID GCA_964442445.

### The Hox gene cluster of P. litorale

Using chelicerate Hox gene sequences [49, 54] as queries we extracted candidate transcripts for nine of the putatively ten ancestral arthropod Hox genes in our *P. litorale* transcriptomes, namely *Labial* (*lab*/*Hox1*), *Proboscipedia* (*pb*/*Hox2*), *Hox3*, *Deformed* (*Dfd*/*Hox4*), *Sex combs reduced* (*Scr*/*Hox5*), *Fushi-tarazu* (*ftz*/*Hox6*), *Antennapedia* (*Antp*/*Hox7*), *Ultrabithorax* (*Ubx*/*Hox8*), and *Abdominal-B* (*AbdB*/*Hox10*). All nine sequences correspond to predicted gene models located on pseudochromosome 56. Subsequent phylogenetic analysis of the extracted protein sequences corroborated our preliminary assignments of Hox gene identities (Additional File 5: Table 4). Thus, we retrieved a single, intact Hox gene cluster spanning over 1.07 Mbp with no evidence for additional Hox clusters or single gene copies on any of the other pseudochromosomes. The nine Hox genes follow the typical ordering (ascending from *Hox1* to *Hox10*) and show identical orientation on the minus strand (Additional File 5: Table 4).

The apparent absence of *Abdominal-A* (*abdA*/*Hox9*) prompted additional analysis. A dedicated search of the genome with the putative but highly divergent partial *abdA* sequence of the sea spider *Nymphon gracile* [55] did not result in any statistically significant hits (also see the “hoxfinder.ipynb” notebook on the GitLab repository under 07-analysis/). Beyond this, also scanning the genome with various chelicerate *abdA* sequences [49, 54] as queries invariably recovered the locus of *Plit-Ubx* (and one instance of *Plit-Antp*; see Additional File 1: Fig. S2, Additional File 6: Table 5), indicating that an *abdA* ortholog is lacking in the *P. litorale* genome (or degenerated beyond recognition). To exclude misassembly as a possible cause for the lack of *abdA*, we additionally used the same collection of chelicerate *abdA* query sequences to scan the deeply sequenced developmental transcriptomes we generated in this study (see Methods for details). After collapsing identical transcripts, we included them in the phylogenetic analysis of chelicerate Hox sequences referenced above. Even though the bona fide *abdA* sequences form only a paraphyletic grade subtending a clade of *Ubx* + *AbdB* in the resulting gene topology, none of the genomic or transcriptomic *P. litorale* sequences fall into this *abdA* group (Additional File 7). Thus, all available evidence unanimously indicates that *abdA* is truly missing in the *P. litorale* genome.

**Fig. 4:**
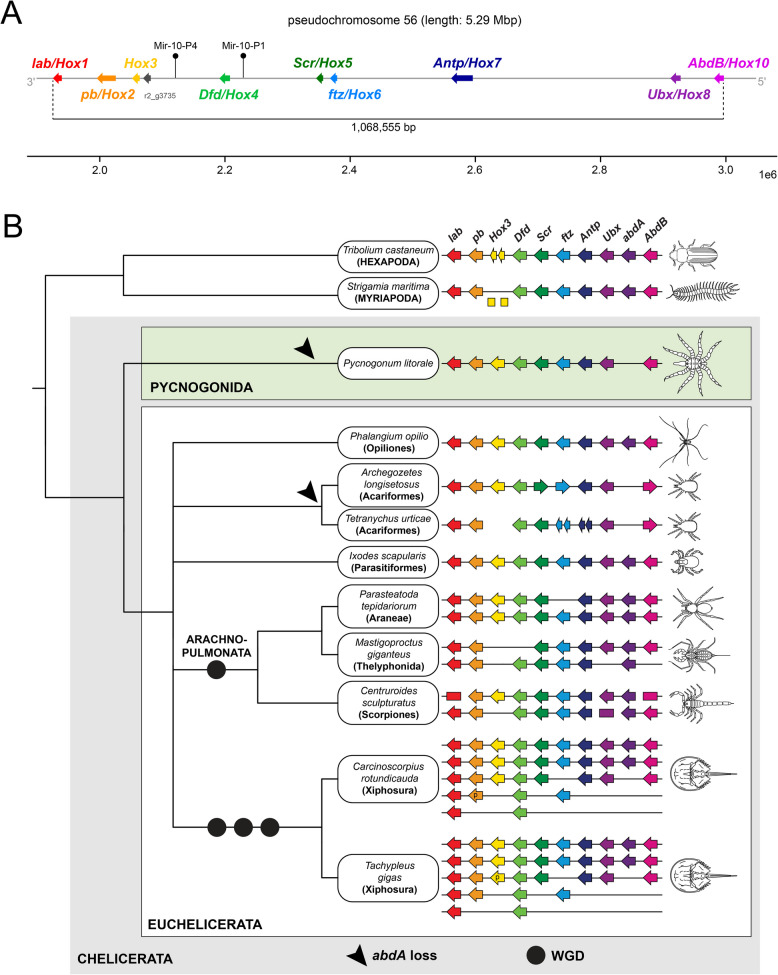
**A** Schematic representation of the *Pycnogonum litorale* Hox gene cluster on pseudochromosome 56, arrows indicate the direction of transcription. Note the absence of *Plit-abdA* between *Plit-Ubx* and *Plit-AbdB*. The positions of the predicted microRNAs Mir-10-P4 and Mir-10-P1 are indicated by black pins. Note the absence of the positionally highly conserved Iab-4 between *Plit-Ubx* and *Plit-AbdB*. **B** Hox gene clusters in chelicerate genomes and in selected outgroups (modified from [56]; supplemented with additional data for *A.longisetosus* [27], *M. giganteus* [49] and *P. opilio* [57]). Arrows represent the direction of transcriptional activity, where known. Circles represent WGD events, with at least two in the lineage leading to extant xiphosurans and another in the stem lineage of Arachnopulmonata. Arrowheads indicate the independent loss of *abdA* in Pycnogonida and Acariformes. p: pseudogene

One minor irregularity in the cluster pertains to the presence of a putative gene between *Plit-Hox3* and *Plit-Dfd* (Fig. 4A). This gene model (r2_g3735) was produced in the second round of BRAKER annotation and a BLAST search against NCBI's nr database finds excellent hits (alignment score ≥ 200) across very diverse taxa (fish, bivalves, sea anemones, sponges, bacteria; see Additional File 8: Table 6). All aforementioned BLAST hits are so-called “hypothetical” or “uncharacterized” proteins without any conserved domains. At the same time, there exist de novo assembled transcripts at the Instar II and Instar V stages that convincingly match to the gene model. Taken together, the evidence suggests that the locus represented by r2_g3735 is a real protein-coding gene of unknown function.

Two of the 70 predicted microRNA genes are located in the Hox cluster. They belong to the MIR-10 family, with MIR-10-P4 (previously named MIR-993) found between *Plit-Hox3* and *Plit-Dfd*, whereas the other paralogue MIR-10-P1 is located between *Plit-Dfd* and *Plit-Scr* (Fig. 4A).

### Other conserved bilaterian homeobox clusters in P. litorale

To further evaluate whether any signs of systematic gene duplications occur in the *P. litorale* genome, we expanded our analyses beyond the Hox genes to other homeobox genes that are arranged in conserved syntenic clusters in (many) bilaterians. This included the NK, NK2, *IRX*, HRO + *Isl*, and SINE clusters, with cluster definitions following [54]. All genes expected in the aforementioned clusters are retrieved in single copies, except for *Msxlx*, which is missing, and *Msx*, *Emx*, and *Hlx*, which have multiple paralogs.

#### HRO (Hbn-Rax/Rx-Otp) + Isl cluster (Additional File 9)

(*Homeobrain-Rax-Orthopedia*) All four genes of the ancestral cluster are represented in single copies in the *P. litorale* gene models. Of them, *Isl* and *Rax* are found together on pseudochromosome 13, while *Otp* lies on pseudochromosome 9 and *Hbn* on pseudochromosome 35.

#### Irx cluster (Additional File 10)

Single orthologs of *Irx1*, *Irx2*, and *Irx3* are present on pseudochromosome 7, forming a syntenic block on the plus strand.

#### SINE cluster (Additional File 11)

Single copies of the three members *Six1/2*, *Six3/6*, and *Six4/*5 are found among the gene models. Being located on different pseudochromosomes (13, 3, and 56 respectively), they do not form a syntenic block in the assembly.

#### NK cluster (Additional File 12)

All genes of the ancestral cluster were found once in the predicted gene models, except for four copies of *Msx* and two copies of *Emx*. The NK cluster is fragmented however—the biggest syntenic block is located on pseudochromosome 6, consisting of *NK5*, *NK1*, *Hhex*, and the four *Msx* paralogs. Another syntenic block, consisting of *NK3*, *NK4*, and *Tlx*, can be found on pseudochromosome 48. The two *Emx* paralogs are located together on pseudochromosome 16. *Lbx* and *NK7* form a fourth syntenic block on pseudochromosome 38. The remaining genes *NK6* and *Noto* are each on different pseudochromosomes.

#### NK2 cluster (Additional File 12)

Single copies of NK2.1 and NK2.2 are positioned close to each other on pseudochromosome 19. Two copies of *Hlx* could be identified on pseudochromosome 17, but their respective nucleotide sequences are identical, hinting at a misassembly. The same subtree contains a nested Dbx clade (Additional File 13). The final NK2 cluster member, *Msxlx*, could not be identified.

Due to the high conservation of the homeobox sequence, additional homeobox genes were retrieved from the *P. litorale* genome and included in the phylogenetic analysis. The calculated gene trees confidently exclude these genes from the syntenic clusters examined here, and we only tentatively identified them via sequence similarity (EggNOG-mapper, BLAST searches against NCBI’s *nr* database).

More details can be found in the GitLab repository [45] under 07-analysis/. The relevant gene models can be found in Additional File 5: Table 4, while the sequences, alignments, and tree files can be found on Zenodo (https://explore.openaire.eu/search/dataset?pid=10.5281%2Fzenodo.14362378).

### Self-synteny analysis

**Fig. 5:**
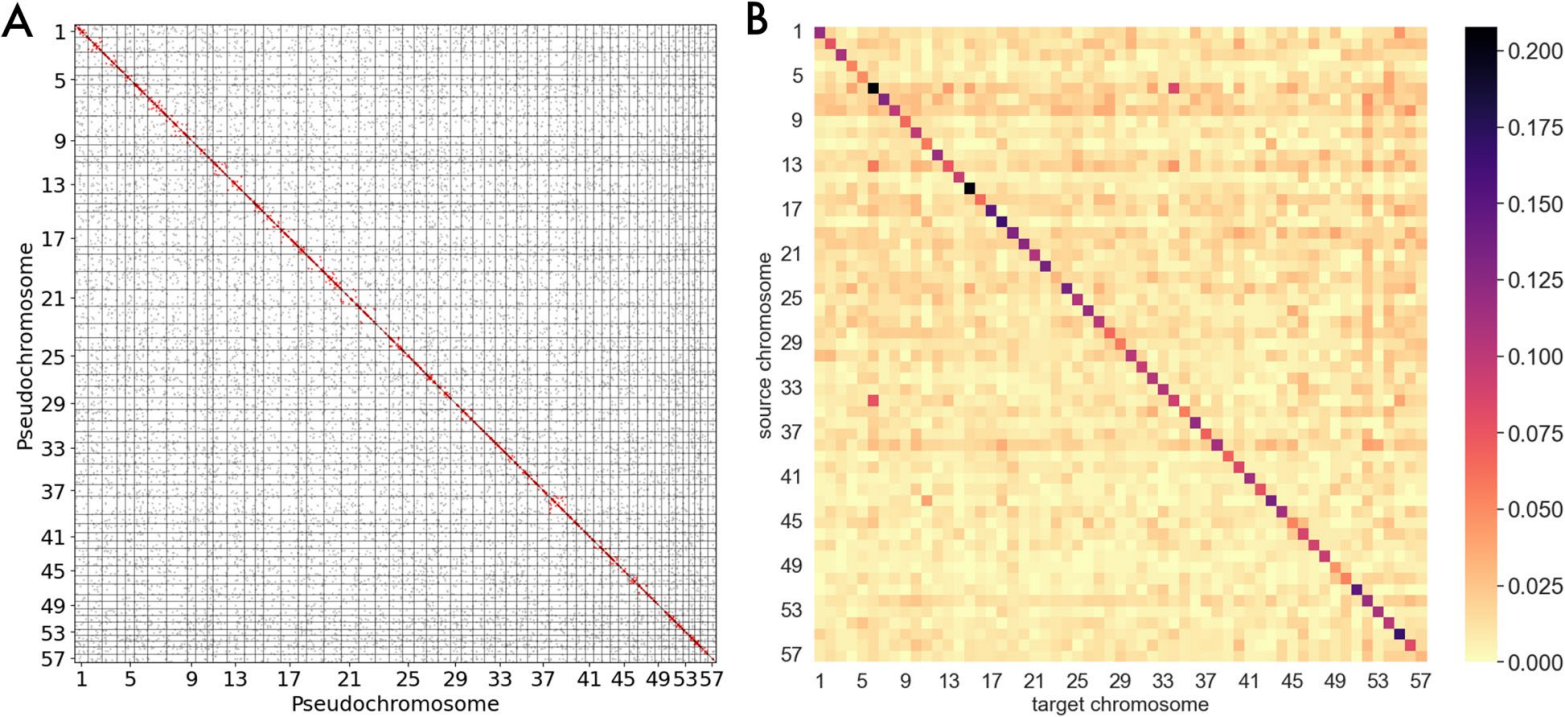
**A** Self-Oxford grid for *P. litorale*. Each point represents an alignment between the predicted peptide sequences for a pair of genes; the alignment covers at least 80% of both peptides’ length at an e-value of 10^−5^ or less. The location of each point is the midpoint between the gene start and end positions on the pseudochromosome. Red points denote matches on the same pseudochromosome; gray points denote matches on different pseudochromosomes. Query sequences are placed on the y-axis, target sequences on the *x*-axis. **B** Heatmap conversion of Oxford grid. Each cell of the matrix corresponds to one cell of the Oxford grid. The color of the cell at position (*x*, *y*) denotes the percentage of genes in chromosome x that have their putative source on chromosome y. The e-value cutoff for paralog detection was 10.^−5^. More details can be found in the corresponding notebook [45] (under 07-analysis/self_synteny.ipynb). The underlying data can be found in Additional File 22: Table 12, also available on Zenodo (https://explore.openaire.eu/search/dataset?pid=10.5281%2Fzenodo.14362378)

We used a modified Oxford grid [58] to identify putative paralogous syntenic blocks within the *P. litorale* genome. Our self-Oxford grid shows no clear pattern in the distribution of putative paralog loci (Fig. 5). Aggregating the total number of putative paralogs per pseudochromosome reveals no obvious outliers (Additional File 1: Fig. S3). On average, the gene pool of each pseudochromosome consists of 20–30% putative paralogs from other pseudochromosomes, depending on the e-value threshold used to define paralogs.

We summarized the Oxford grid by examining each pair of pseudochromosomes separately and counting the number of putative paralogs between them, normalized by the total gene count of the “donor” pseudochromosome (Fig. 5). For each pseudochromosome, on average, every other pseudochromosome contributes 1.8% of the gene pool (minimum: 0%, maximum: 11.6%). These values decrease with higher e-value thresholds, reaching 1.1%, 0% and 8.4%, respectively, for an e-value of 10^−60^.

## Discussion

### The *P. litorale* genome in an arthropod context

Genomic resources for Chelicerata are quite asymmetrically distributed, with gaps in high-quality datasets for several understudied orders [59]. Prior to our study, a single pycnogonid draft genome of *Nymphon striatum* had been publicly available [31]. The small body size of this species led these authors to pool multiple (40) individuals, increasing the effective heterozygosity. This affected the quality of the final assembly that after scaffolding with mate-pair reads had an N50 of 701.8 kb only, despite starting from PacBio HiFi reads with an N50 of 19.4 kb. By comparison, our use of a single female of *P. litorale* as input material yielded a scaffold N50 an order of magnitude higher (7.97 Mb), despite starting with ONT reads with an N50 four times lower (5.3 kb). Even though both genomes have comparable BUSCO completeness scores (96–97% BUSCO Arthropoda), the *P. litorale* genome thus exhibits a significantly higher contiguity compared to the *N. striatum* assembly, reaching near chromosome level.

The number of 57 pseudochromosomes in the *P. litorale* assembly is high for chelicerates, although more than twenty chromosomes are not unprecedented in arachnid taxa [60, 61] and even higher numbers are known for some other aquatic arthropods, such as decapod crustaceans [62–65] (Additional File 1: Fig. S4A). While experimental confirmation via karyotyping has not so far been pursued, the high BUSCO scores point toward a fairly complete genome, similar to other arthropods (Additional File 1: Fig. S4B). The size of the assembly lies in the previously reported range for chelicerates, albeit on the shorter side (Fig. 3A), with the only genomes that are consistently smaller being those of mites [66]. The number of predicted protein-coding genes of *P. litorale* is modest compared to most other chelicerates (Fig. 3B), following the expected correlation with genome size (Fig. 3D).

Current chelicerate genome data suggest that they can be highly repetitive, with values routinely over 50–60% [47, 67] (Figs. 2 and 3C). In this sense, the predicted repeat content of the *P. litorale* genome presented here (61.05%) is much more in line with what is already known from chelicerates than the *N. striatum* draft genome, which at just 7.14% is a clear outlier even in the context of all arthropods. However, while the *P. litorale* genome size and total repeat content are, on their own, within previously reported ranges for chelicerates and arthropods in general, the combination of high repeat content (> 50%) in a small genome (< 500 Mb) is not (Fig. 3F). By demonstrating the frequent presence of repeat families in raw reads, we could exclude contamination as a likely source of the repetitive sequences, indicating that the remarkably high repeat/genome size ratio in *P. litorale* is real. Future studies will have to elucidate whether this represents a shared genomic feature of the entire pycnogonid lineage.

It is well-established that transposable elements (TEs) are potent drivers of genome evolution, offering a mutation source, providing raw material for novel cellular genes, enhancing rearrangements, duplicating or shuffling genes, and dispersing regulatory elements to new loci, among other functions [68, 69]. Moreover, there is a growing body of evidence that horizontal transfer of TEs between distantly related eukaryotes with close interactions (e.g., predator–prey, parasite-host) is more common than previously thought [70–72]. Given that *P. litorale* is an obligatory, tissue-sucking micropredator on selected sea anemone species, our cursory identification of similar repetitive motifs shared in the genomes of *P. litorale* and *M. senile* thus warrants closer inspection and characterization of the unclassified repeats in the context of potential TE transfer.

Prior to our study, the microRNA families Mir-3931, Mir-5305, and Mir-5735 were classified as chelicerate-specific [73]. With data for pycnogonids lacking, however, only euchelicerate taxa could be included in previous microRNA surveys, leaving the question unresolved whether these three families evolved already at the base of the chelicerate tree. Interestingly, we found only evidence for Mir-3931 s in the *P. litorale* genome. As the high score of > 95% for conserved microRNAs speaks for a good representation of the total microRNA content in the assembly, this suggests that only Mir-3931 was present in the last common ancestor of Chelicerata, whereas Mir-5305 and Mir-5735 are likely derived microRNAs of Euchelicerata.

### Novel genomic and transcriptomic resources unlock the P. litorale for chelicerate evo-devo

Molecular data for sea spiders have so far been scarce and taxonomically fragmented, hampering the interrogation of the pycnogonid body plan and its development with modern approaches. Until recently, gene expression data were limited to three Hox genes in *Endeis spinosa* larvae [74], while sequence resources were restricted to a draft genome for *Nymphon striatum* [31] and to bulk transcriptomes for a handful of other species [7]. By sequencing the *P. litorale* genome and generating stage-specific transcriptomes (Additional File 2: Table 1), we provide here the most complete molecular description of pycnogonid development to date. This combines synergistically with pre-existing knowledge on laboratory husbandry [10, 39], ontogeny and adult morphology (e.g., [36–38, 75]), as well as recently established HCR-FISH protocols in *P. litorale* [41]. Together, this resource expansion enables state-of-the-art studies on pycnogonids, paving the way to a better understanding of the evolution, development, and general biology of this ancient and phylogenetically important chelicerate lineage. Among others, the indirect developmental mode of pycnogonids promises new insight into the molecular underpinnings and evolution of chelicerate body patterning, which so far largely relies on a few arachnid species (e.g., [76, 77]) that exhibit direct development as one of the common, derived adaptations of terrestrialization. Beyond this, the high-quality genome of *P. litorale* represents an important milestone toward unlocking novel macrosynteny-based phylogenetic approaches (e.g., [78]) for the interrogation of the recalcitrant higher-order relationships of Chelicerata.

### The P. litorale genome provides evidence against a WGD in the chelicerate ancestor

Whole-genome duplications abruptly expand the genetic material of a species, increasing the chances for sub- or neo-functionalization of protein-coding genes, and providing new regulatory possibilities, either through the modification of existing gene regulatory networks or the emergence of novel ones. While most paralogs created by WGD are soon lost, such dramatic evolutionary events inevitably leave a trace in the affected genomes [79]. In the widely accepted vertebrate case, two rounds of WGD were first hinted at by the discovery of multiple Hox gene clusters [80], received support by the discovery of syntenic blocks of paralogous genes [81], and were finally confirmed by genome-wide studies of paralog abundance and synteny on multiple vertebrates [82].

Corresponding to vertebrates, similar signatures of WGD have been observed in some chelicerate taxa, namely xiphosurans and arachnopulmonates (e.g., [23, 24, 26, 49, 56]. Accordingly, the occurrence of WGD events in the evolutionary history of chelicerates is now widely accepted, with only few recent studies still questioning this view (see [83] for arachnopulmonates). However, due to the partially unresolved chelicerate phylogeny (e.g., [4]), temporal resolution and polarization of WGD events as well as the reconstruction of the ancestral chelicerate condition are still problematic. To help address these issues, we pursued two lines of inquiry regarding WGD in first high-quality genome of a sea spider, representing the sister group of all other chelicerates: we identified putative members of homeobox gene families with conserved syntenic relationships, and performed a genome-wide paralog synteny analysis.

We examined members of several conserved homeobox gene clusters (Hox, NK, NK2, SINE, HRO, *Irx*) previously scrutinized in the context of WGD in chelicerates (e.g., [24, 49, 54, 83]. In all cases, we find no evidence of systematic duplications in *P. litorale*. The Hox, HRO, SINE, and *Irx* clusters contain one ortholog of each gene member, except for the lack of *abdA* in the Hox cluster (see next section for in-depth discussion of *abdA*). The NK2 cluster misses *Msxlx.* Only the fragmented NK/NK2 clusters show, next to single orthologs of most gene members (*NK1*, *NK2.1, NK2.2, NK3, NK4, NK5, NK6, NK7, Hhex, Lbx, Tlx, Hlx,* and *Noto*), two instances of gene duplications, with four paralogs for *Msx* as well as two paralogs for *Emx*. However, our phylogenetic analysis clearly supports lineage-specific tandem duplications as the underlying evolutionary cause; furthermore, the gene models for all four *Msx* paralogs and both *Emx* paralogs are located in close proximity on their respective pseudochromosomes, lending further support to the tandem duplication hypothesis. The *Hlx* case is a bit more complicated, but as the nucleotide sequences are identical over the length of the entire gene model, the duplication appears to be an assembly artifact. In the absence of additional evidence, it was not clear which gene model should be kept, so both copies were marked accordingly in the genome annotation file.

Similarly, we explored the *P. litorale* genome for signs of WGD using a modified Oxford grid where we plotted the coordinates of putative paralogous loci on the *P. litorale* genome instead of orthologous loci on two related genomes. This (visual) self-synteny analysis revealed no striking syntenic blocks of duplicated loci. Instead, the Oxford grid shows an almost uniform, sparse spread of putative paralogs across the assembly’s pseudochromosomes. To ensure that sequence similarity thresholds were not responsible for this result, we introduced more stringent e-value cutoffs, but the result did not change in a meaningful way.

To examine the results in a more quantitative manner, we converted the Oxford grid to a matrix by comparing each pair of pseudochromosomes and counting the number of putative paralogs between them, normalized by the total gene count of the “acceptor” pseudochromosome. In the case of an underlying WGD, we would expect this matrix, when visualized as a heatmap (Fig. 5) to show strong off-diagonal signal connecting the duplicated pseudochromosomes. However, no such signal is evident. Instead, with increasing stringency in the paralog definition (higher e-value thresholds), the already strong contrast between the diagonal and the rest of the matrix becomes even stronger. Indeed, most of the putative paralogs seem to originate from the pseudochromosome itself, something that would be expected if tandem duplications were more common than large-scale chromosome rearrangements.

Accordingly, even though our work does not substitute a formal synteny and presence/absence analysis in the vein of [78, 82], it presents accumulated evidence against a WGD in the first high-quality genome for a sea spider, anchoring a non-duplicated genome in the chelicerate ground pattern (Fig. 4B). This unequivocally polarizes the non-duplicated genomes of apulmonate euchelicerate taxa as the ancestral condition and further corroborates that the xiphosuran and arachnopulmonate WGDs are derived [25, 54].

### The reduced pycnogonid opisthosoma and the lack of abdA—cause or effect?

The ancestral arthropod Hox cluster was likely comprised of ten genes [84], which contribute to governing segment identity along the anterior–posterior axis during development. So far, data on pycnogonid Hox genes have been fragmentary. The earliest survey of two species (*Nymphon gracile*, *Endeis spinosa*) was based on polymerase chain reaction (PCR) and did not retrieve a full Hox gene complement for either sea spider [55, 74], although, taking both species together, orthologs of all ten genes were present. While *abdA*/*Hox9* was not found in *E. spinosa*, only a highly divergent *abdA* sequence was identified in *N. gracile*. However, the draft genome of *N. striatum* [31], a congener of *N. gracile*, shows no evidence of *abdA*, and neither do any of the hitherto generated developmental transcriptomes of other pycnogonid species [7]. In this study, we screened a comprehensive series of stage-specific developmental transcriptomes of *P. litorale* and report its completely sequenced Hox cluster, the first for a sea spider (Fig. 4A). Given the lack of any *abdA* orthologs in our transcriptomic data and its absence from the intact Hox gene cluster, our results provide strong genomic evidence for a degradation/loss of *abdA* in *P. litorale.* This inference receives additional support from the lack of Iab-4 in our microRNA survey. Together with Mir-10-P4 and Mir-10-P1, Iab-4 belongs to a conserved trio of microRNAs associated with the arthropod Hox cluster, in which Iab-4 is almost invariably located between *abdA* and *AbdB* (e.g., [84, 85]). Accordingly, the absence of a recognizable Iab-4 from the otherwise very complete suite of conserved microRNAs serves as additional indication for significant sequence degradation in the Hox cluster region that typically includes *abdA*. These novel findings in *P. litorale* call for a reinvestigation of the *N. gracile* Hox gene cluster with state-of-the-art sequencing approaches, as well as new genomic resources for Austrodecidae, the putative sister group of the remaining Pycnogonida [7], to test the inference that *abdA* loss occurred already at the stem of the sea spider crown group.

The posterior Hox gene *abdA* is typically expressed in major parts of the chelicerate opisthosoma [15, 86], and it was previously hypothesized that the reduction of the pycnogonid opisthosoma may be linked to the loss or strong degeneration of this gene, leading to a loss of its patterning function [55]. Intriguingly, the same correlation has been noted for certain acariform mites with extremely reduced opisthosoma [27, 30, 84] (Fig. 4B), and, outside Chelicerata, in cirripede crustaceans, which likewise exhibit an extreme reduction of their posterior tagma, the abdomen [85, 87]. Losses of multiple trunk Hox genes, including *abdA*, are also associated with the compaction of the tardigrade body plan [88]. Accordingly, the correlation of posterior tagma reduction and the absence of *abdA* across evolutionarily distant arthropod lineages may hint toward a second, higher-order function of *abdA* beyond specification of posterior segment identity, namely the formation and maintenance of posterior body segments. A precedent for such dual functions of Hox genes is known for *labial*/*Hox1* orthologs, which not only convey identity to the tritocerebral segment in insects and spiders, but are also crucial for its maintenance [89, 90]. If a similar dual function were to apply to *abdA*, its loss may have been sufficient to cause the reduction of posterior segments. In line with this scenario, an abrupt removal of *abdA* from the Hox cluster has been hypothesized for the body plan evolution of acariform mites [84]. This was based on the reversed orientation of *AbdB*/*Hox10* compared to the rest of the genes in the Hox cluster (Fig. 4B), hinting at a potential chromosome inversion that impacted part of the posterior Hox genes.

Ideally, this proposed relationship would need to be tested via functional interrogation of *abdA*, something that has been hitherto unsuccessful in chelicerates [56]. Outside of Chelicerata, however, functional insights were gained from several insects and one malacostracan crustacean, where *abdA* clearly instructs segment identity, whereas there is no evidence of its involvement in posterior segment formation and maintenance comparable to *lab* [91–94]. By extrapolation, this suggests that the loss of *abdA* function may also in chelicerates represent an insufficient condition for opisthosomal segment reduction. Moreover, unlike in acariform mites, the Hox cluster of *P. litorale* displays uniform orientation of all nine Hox genes present (incl. *AbdB*), disfavoring the idea of a chromosome inversion event that removed *abdA* from the cluster during sea spider evolution. Taken together, these observations suggest that the causal relationship may rather be reversed: the reduction of opisthosoma segments rendered their further specification obsolete, making a loss of *abdA* possible, for instance due to mutation accumulation to the point of extreme degradation and eventual pseudogenization. Notably, a similar scenario has been recently proposed to underlie the evolution of the compacted tardigrade body plan, which correlates with the absence of multiple Hox genes [95].

At the morphological level, fossils provide some support that the compaction of the segmented opisthosoma may have resembled a gradual process in the pycnogonid stem lineage (e.g., [96, 97]), suggestive of incremental instead of abrupt changes in the underlying developmental programs. However, it remains unknown to what extent crown group pycnogonids may have retained vestigial posterior segments during their development. While the transitory presence of up to two small posterior ganglia has been taken as evidence for this phenomenon [40, 98], it awaits further confirmation by expression studies on the gene regulatory networks governing segmentation and segment identity in advanced postembryonic instars of *P. litorale*. Similar to corresponding studies on acariform mites and cirripedes [99–102], such experiments will help clarifying whether the debated pycnogonid anal tubercle evolved by fusion of several vestigial opisthosomal segments, represents the terminal telson only, or is a composite structure of both elements [5, 10, 40, 98].

## Conclusions

In this study, we generated the first high-quality genome and a comprehensive series of stage-specific developmental transcriptomes for a sea spider, providing important reference points for studies into chelicerate evolutionary developmental biology and arthropod genome evolution. These novel resources will reinvigorate the systematic examination of pycnogonid development at the molecular level and afford the opportunity to polarize evolutionary developmental trajectories of body patterning at the base of the chelicerate tree. Investigation of several conserved homeobox gene clusters and self-synteny analysis of the *P. litorale* genome provide genomic evidence that no WGD events occurred already in the chelicerate stem lineage. Beyond this, the first completely annotated Hox gene cluster of a sea spider corroborates a lack of *abdA*, highlighting the independent evolutionary recurrence of a common genomic motif in distantly related arthropod groups that share a significant reduction of their respective posterior tagma.

## Methods

### Animal collection and husbandry

The *P. litorale* specimens used for genome and RNA sequencing originated from two different geographic localities: The first draft genome assembly is based on a single adult female from our *P. litorale* culture at the University of Vienna, Austria (specimen H1). This laboratory population derives from animals collected during low tide in the rocky intertidal of Helgoland in the North Sea (see [10] for details on subsequent husbandry). All developmental stages (embryonic stages, postembryonic instars I–VI, first juvenile instar and subadult; see [37–39] for definitions) used for RNA-seq were reared and collected from the same in-house laboratory population.

For Omni-C and PacBio sequencing, *P. litorale* specimens were collected in the Gulf of Maine off the US East coast under rocks in tide pools at low tide by Tim Sheehan (44° 54′ 03.5″ N 67° 07′ 17.8″ W, Pembroke, Maine, USA) and transported to Madison, Wisconsin. This wild population constitutes the source of previously published transcriptomic data for *P. litorale* (adult stage) [103]. One male specimen (specimen M1) was used for PacBio sequencing and the second (specimen M2) for Omni-C sequencing.

We confirmed species identity of the two populations via cytochrome *c* oxidase subunit I (COX1) barcoding with widely used standard primers [104]. Obtained sequences were aligned and compared with corresponding barcodes of our in-house laboratory population and of additional *P. litorale* sequences deposited in the NCBI repository. Sequence identity ranged from 98.6 to 100% across all samples included in the alignment (Additional Files 14, 15). Between the Gulf of Maine specimens and the laboratory population from Helgoland, sequence identity was even higher (99.5–100%), confirming the widely accepted trans-Atlantic distribution of *P. litorale* previously inferred from morphological species determination (e.g., [105]).

A detailed table of all BioSamples involved in this study can be found in Additional File 2: Table 1.

### High molecular weight (HMW) DNA extraction and sequencing

#### ONT long-read sequencing

An adult female (specimen H1) was separated from the other animals and starved for a week in a small cage inserted in our seawater system without access to prey. Prior to DNA extraction, the animal was repeatedly rinsed in filtered artificial seawater (32‰, Red Sea, Red Sea Fish Pharm LTD) to minimize contamination by non-target DNA from externally attaching microorganisms. A Qiagen MagAttract HMW DNA kit (Cat. No. 67563) was used for DNA extraction following the manufacturer’s protocol, with the entire animal serving as input tissue. For this purpose, the female was placed in a Petri dish, cooled down on ice, quickly cut into several pieces using sterile microsurgical scissors and immediately transferred into the lysis buffer. After brief manual homogenization with a sterile pestle, the tissue pieces were incubated overnight at 56 °C under constant shaking at 900 rpm. The HMW DNA was eluted in 100 µL AE buffer (10 mM Tris–HCl, 0.5 mM EDTA, pH 9.0).

The HMW DNA was subsequently transferred to the Vienna BioCenter (VBC) sequencing facility, where library prep and sequencing took place. Sequencing was performed on a PromethION machine. Reads were basecalled with the GPU-version of Guppy v6.5.7 (minimap2 v2.24-r1122 [106]), using the r10.4.1 basecall model. Quality control was performed with nanoplot [107].

#### PacBio HiFi long-read sequencing

Specimen M1 was maintained at 8 °C without food for 11 days to minimize gut content contamination. High molecular weight DNA was extracted using the Qiagen MagAttract HMW DNA kit, following the manufacturer’s instructions. After cooling on ice, the entire male was used as input tissue, with sectioning of live tissue with a sterile razor blade and immediate transfer into lysis buffer. After brief manual homogenization with a sterile pestle, the tissue pieces were incubated for 5 h at 56 °C with manual perturbation every 45–60 min, until the buffer appeared translucent. HMW DNA was eluted in 100 µL AE buffer. The DNA-binding magnetic beads were incubated in another 100 µL AE buffer overnight and eluted the following day. Samples were submitted for sequencing at the UW-Madison BioTechnology Center core facility on a PacBio Sequel II machine, using the standard manufacturer’s protocols for the Sequel II Sequencing Kit 2.0. The library was sequenced on 1 SMRT Cell (8 M) in CCS mode for 30 h. Analysis was performed with SMRT Link v10.1 software, requiring a minimum of three passes for CCS generation.

### Omni-C sequencing

Specimen M2 was maintained at 8 °C without food for 11 days to minimize gut content contamination and flash frozen with liquid nitrogen. The whole specimen was immediately transferred to dry ice and submitted for library preparation by Cantata Bio (Scotts Valley, CA, USA) with the Dovetail Omni-C library kit. Tissue from nearly the whole specimen was used to generate the Omni-C library. Chromatin was fixed in place with formaldehyde in the nucleus. Fixed chromatin was digested with DNase I and then extracted, chromatin ends were repaired and ligated to a biotinylated bridge adapter followed by proximity ligation of adapter containing ends. After proximity ligation, crosslinks were reversed and the DNA purified. Purified DNA was treated to remove biotin that was not internal to ligated fragments. Sequencing libraries were generated using NEBNext Ultra enzymes and Illumina-compatible adapters. Biotin-containing fragments were isolated using streptavidin beads before PCR enrichment of each library. The library was sequenced on an Illumina HiSeqX platform to produce ~ 30 × sequence coverage.

### RNA extraction and short-read sequencing

Embryonic stages, postembryonic instars I-VI, the first juvenile instar and subadults of *P. litorale* were separately transferred into Eppendorf tubes containing RNAlater (Sigma-Aldrich, Cat. No.: R0901) at ambient temperature. After the specimens had settled to the bottom, the tubes were left overnight at 4 °C and subsequently stored at − 20 °C until further processing. Extraction of total RNA was performed with a Qiagen RNeasy Plus Mini Kit (Cat. No.: 74134) according to the manufacturer’s protocol, eluted in 30 µL RNase-free water and stored at − 70 °C until sequencing. The RNA was transferred to the VBC sequencing facility, where library prep and sequencing took place. The libraries were sequenced on half of a NovaSeq S4 lane (300 cycles). The sequencing data was basecalled with RTA v3.4.4 and demultiplexed by bcl2fastq2 v2.20.0 with default parameters by the VBC sequencing facility.

Zygotes, early cleavage, and more advanced embryonic stages from our in-house colony were also sequenced independently in Madison, Wisconsin. The samples were preserved and transported in tubes containing RNAlater and extracted using TRIzol reagent (Thermofisher, Cat. No.: 15596026) following the manufacturer’s instructions, with elution into THE RNA Storage Solution (Thermofisher, Cat. No. AM7001). Library preparation with Illumina TruSeq library kits was performed at the UW-Madison BioTechnology Center. Paired end 2 × 150 bp sequencing was performed on an Illumina NovaSeq platform.

For an overview of samples and sequencing depth, please refer to Additional File 2: Table 1.

### Full-length mRNA sequencing

Prior to enzymatic shearing, small volumes of the same RNA extractions used for short-read RNA sequencing were mixed to obtain an RNA pool covering all phases of development (Additional File 16: Table 7). The pooled sample was subsequently transferred to the VBC sequencing facility for long-read mRNA sequencing (Iso-seq) on a PacBio platform, where it was multiplexed with samples from two other species.

The data were processed according to the instructions outlined in the publicly available documentation from PacBio [108]. Briefly, subreads were processed to circularized consensus sequences with ccs v6.4.0; the CCS were demultiplexed (separating the samples) and primers were removed with lima v2.9.0; the resulting BAM files were refined by trimming poly(A) tails and removing concatemers with the refine command of the isoseq package (v4.0.0); the refined transcripts were clustered with the cluster2 command of the same package; the bam2fastq utility (v3.1.1) was used to convert the BAM files to FASTQ format; finally, the clustered transcripts were mapped to the genome with pbmm2 (v1.13.1) and the result was used to collapse the refined CCS reads with the collapse command of the Iso-seq package and obtain a GFF file. More details can be found in the GitLab repository for this study [45] under 05-transcriptomes/.

### Genome assembly, scaffolding, and contaminant filtering

To estimate genome size and heterozygosity, we quantified frequency spectra for k-mers of size *k* = 21 with jellyfish v2.3.0 [109] and analyzed them on the GenomeScope [43] and GenomeScope2 [44] webservers. This approach is intended for very highly accurate short reads; therefore, the analysis of the long-read data should be viewed with caution, but with high enough coverage the k-mer spectra of error-prone reads should be approximating the true distributions.

The PacBio data (specimen M1) had a k-mer coverage of 8–17 × according to GenomeScope (Additional File 1: Fig. S1A, C), below the theoretical 30 × threshold that was often suggested in the short-read era (Sims, 2014). Nevertheless, we tried an assembly with Flye v2.9.2 [110], using default parameters. The ONT reads (specimen H1) had a 31 × k-mer coverage. We tested Flye v2.9.2, shasta v0.11.1 [111], and Verkko v2.0 [112], all with default parameters. After comparing the assemblies for completeness and contiguity, we proceeded with the one generated by Flye.

To scaffold, we followed the “HiC_map7” pipeline from Schultz, [113], adapting it to our computing environment. We mapped the omni-C data onto the assembly using chromap v0.2.6-r490 [114]. The resulting SAM file was sorted and indexed with samtools v1.16.1 (using htslib 1.16) [115], before being passed on to yahs v1.2a.2 [116]. The result was edited manually using juicebox v2.15 [117, 118].

All the scripts for genome assembly, quality control, and evaluation can be found in the GitLab repository [45] under 01-assembly/.

We used MMseqs2 v6f45232 [119] to download the UniRef90 database [120], index the draft genome for alignment, and align the draft genome against UniRef90, adding the taxonomic information of each hit. We summarized the results by aggregating the hits within each scaffold according to their taxonomic assignment. We then removed scaffolds where less than 90% of hits were of metazoan origin. This filtering marked and removed pseudochromosomes 52 and 53 as contaminants. However, we initially proceeded without renaming the following scaffolds, to be able to follow potential issues back to their original source. After the annotation procedure was finished, we used the Linux bash utility sed to rename pseudochromosomes 54–59 to 52–57, respectively, in the genome sequence (FASTA) and annotation (GFF) file. Bash scripts and Jupyter notebooks for the individual steps can be found in the GitLab repository [45] under 04-contam/.

### Genome annotation

We used RepeatModeler v2.0.5 [121] to build a repeat family database for *P. litorale*, running it with the -LTRStruct argument to also characterize long terminal repeats. The families predicted by RepeatModeler were then used by RepeatMasker v4.1.6 to softmask the draft genome.

We used BRAKER v3.0.6 [52] with the short-read transcriptomic data and the Arthropoda OrthoDb v11 [122] protein sequences to predict protein-coding gene models on the softmasked draft genome. We used the default Augustus configuration directory, copied from a local clone of the Augustus repository (commit d0b1b6c, Nov. 27, 2023). We used the containerized version of BRAKER, run with Singularity v3.8.6. To speed up calculations, the transcripts were mapped to the draft assembly with STAR v2.7.11b [123] before running BRAKER. More details can be found in the GitLab repository [45] under 06-annotation/.

We merged the GFF file produced from BRAKER with the one produced from Iso-seq collapse (see Methods) with the “intersect” program of the bedtools suite (v2.30.0) [124] and used custom Python code to analyze the overlap [45] under 06-annot/annot1-isoseq_confirm.ipynb. We tested how many Iso-seq clusters (interpreted as genes) were overlapping with each BRAKER gene model. We considered two cases of significant overlap for gene models and clusters that occupy similar loci on the same strand: first, if one gene model only contained transcripts from the same cluster, we asked that at least 40% of the transcripts either contained the full gene model or were fully contained within it. If that was not the case, we demanded that the average overlap between transcripts of the cluster and the gene model exceeded 50%. These cutoffs ensured that we did not merge a gene model with a cluster in cases of partial overlap (e.g., the 5-prime end of the gene model overlaps with the 3-prime end of the cluster). We generated a merged GFF file that retained the unique BRAKER gene models and Iso-seq clusters and reconciled the overlapping regions. Gene models deriving from the Iso-seq clusters were given the cluster name (“PB.number > ”); gene models deriving from BRAKER kept their BRAKER name (“g < number > ”).

We then used bedtools intersect with the -v flag to exclude from the RNA-seq data everything mapping to loci covered by the merged GFF. The reduced RNA-seq data were then supplied to BRAKER and a second round of gene model prediction was run with the same parameters as the first. Using bash command line tools and bedtools intersect, we extracted the exons from the round 2 GFF file and cross-referenced them with the merged GFF file; we used this information to extract novel gene models from the round 2 GFF file. These were then deposited in a new GFF file with the agat_convert_sp_gxf2gxf.pl script (AGAT suite) and appended to the merged GFF. To avoid confusion, novel gene models kept their BRAKER-style IDs but with a prefix (“r2_g < number > ”).

In a third step, we performed de novo assemblies for the deeply sequenced RNA-seq datasets (see Additional File 2: Table 1) with Trinity v2.15.1 [125], with the –trimmomatic and –no_salmon flags. We extracted complete open reading frames (ORFs) from the de novo transcriptomes with TransDecoder v5.7.1 [126], mapped them against the draft genome with minimap2 v2.28, and exported the mapping results in GFF format with the agat_convert_minimap2_bam2gff.pl script from the AGAT suite (v1.4.0) [127]. We then used bedtools intersect with the -v flag to extract map events that did not overlap with the already annotated gene models (merged + round2 GFF). We then used bedtools to find the pairwise intersections of the resulting GFFs with the instar III-based GFF. The resulting overlap table was analyzed with custom Python code [45] (under 06-annotation/) and the overlaps were reconciliated into gene models, which were given ascending unique IDs similar to Trinity transcript IDs (“DN < number > ”) but with a prefix to denote their origin (“at_DN < number > ”, for “assembled transcriptome”). The result was written out in GFF format and concatenated to the merged + round2 GFF file. At this step, any remaining overlapping gene models were removed. Finally, the merged GFF was sorted and formatted with genometools gff3 v1.6.2 [128], producing the final protein-coding gene annotation. The entire process is described in depth in the GitLab repository [45] under 06-annotation/. Finally, we annotated tRNA genes with tRNAscan-SE v2.0.12 [129].

### Orthology assignment

We used the possible ORFs and agat_sp_extract_sequences.pl script from the AGAT suite (v1.4.0) to extract exons, using the draft genome and the final GFF file as input. The result was processed with TransDecoder v5.7.1 to produce a list of the most probable transcripts. The TransDecoder-predicted peptide file, containing multiple entries per gene, was submitted to the EggNOG-mapper server (v2.1.12) [130] and mapped against version 5 of the EggNOG database [131] using default parameters. The results were downloaded and analyzed further with a Python notebook [45] (under 07-analysis/emapper_output.ipynb). To improve usability, annotations of different transcripts and isoforms were collapsed for each gene ID by keeping the entry with the highest bit score, leading to a slimmer look-up table.

### MicroRNA survey

For the prediction of the conserved microRNA complement and in the absence of small RNA sequencing, the genome was subjected to MirMachine [132] analysis, using protostome models and “Chelicerata” as search node based on MirGeneDB 3.0 annotations [73]. High and low confidence predictions were compared to the expected microRNA complement.

### Hox gene cluster annotation

To identify *P. litorale* Hox genes, publicly available Hox gene sequences from the sea spiders *Nymphon gracile* and *Endeis spinosa* were downloaded from NCBI [133]. In addition, the Hox gene sequences of the harvestman *Phalangium opilio*, an apulmonate representative with a well-annotated, unduplicated Hox gene complement [29, 57], were used as queries in BLAST searches (tblastn) against our different *P. litorale* transcriptomes. The best *P. litorale* results (complete CDS) were used as queries in BLAST searches (blastx) against the NCBI database for preliminary confirmation of the assignment of Hox gene identity. The putative *P. litorale* Hox gene sequences were then used to scan the genome with MMSeqs2 using default parameters [45] (under 07-analysis/genes.sh). The predicted peptides of identified genomic loci were extracted with TransDecoder v5.7.1 [126].

For the posterior Hox gene *Abdominal-A* (*abdA/Hox9*), we performed an extended, more targeted search against the draft genome (MMseqs2 with default parameters). The query sequences were taken from [49], who extend a previous comprehensive study by [54] with the vinegaroon *M. giganteus* and the harvestman *O. spinosus*. The same query sequences were used to scan our deeply sequenced stage-specific developmental transcriptomes (embryonic morphogenesis stage 3–4, Instar I-VI, juvenile instar I, subadult; for stage nomenclature refer to [37, 39]).

To confirm our preliminary *P. litorale* Hox gene identification, we aligned the homeobox sequences from [49], the putative genomic Hox gene loci of *P. litorale*, and all transcripts extracted from our transcriptomic resources by *abdA* screening, using mafft v7.526 [134] with default parameters. The alignment was manually inspected to exclude misalignment and trimmed with Jalview [135] to only contain the homeobox domains. Homeobox domains from NK-family genes (*C. sculpturatus* NK1-B-1, *D. melanogaster* slou, *L. polyphemus* Slou D-a, *P. opilio* NK1, *P. tepidariorum* NK1-B-1, *S. maritima* NK1, *T. castaneum* Slou, *T. tridentatus* Slou A) were added as outgroups, and the sequences were realigned with mafft in –localpair mode and a maximum of 1000 iterations. The resulting alignment was used to calculate a maximum likelihood tree with IQ-TREE v2.3.6 [136], using automatic model finding [137] and 1000 ultrafast bootstrap iterations [138]. The resulting gene tree (best-fit model: Q.insect + R3) was analyzed with TreeViewer v2.2.0 [139].

More details can be found on the GitLab repository [45] under 07-analysis/hoxfinder.ipynb.

### Analysis of additional conserved homeobox gene clusters

We compiled a list of additional arthropod homeobox sequences from genes belonging to the conserved bilaterian NK, NK2, HRO + *Isl*, and SINE gene clusters from a recent comprehensive spider homeobox gene analysis [54]. We used this list to scan against the *P. litorale* genome using mmseqs easy-search. The top hits (e-value < 10^−20^) were mapped to gene models, and the predicted peptides were extracted with TransDecoder. For the genes of each cluster, multiple sequence alignment and phylogenetic analysis was subsequently performed as described for the Hox genes. The best fit models chosen for each cluster were as follows: for the HRO + *Isl* cluster, Q.insect + G4; for the SINE cluster, Q.plant + G4; for the *Irx* cluster Q.insect + F + I + R5; for the NK and NK2 clusters, Q.insect + I + G4.

The *Irx* gene cluster presents additional challenges, since the homeobox sequences are conserved to a very high degree [140]. Therefore, we used the full-length sequences provided by [54] as queries to scan the *P. litorale* genome. We mapped the loci of high-quality hits (e-value < 10^−20^) to gene models and retrieved their predicted peptide sequences. After alignment with mafft v7, manual inspection revealed that multiple candidate sequences did not have a conserved homeobox domain. These sequences were queried against the NCBI nr database and returned exclusively non-transcription factor, non-homeobox hits, and therefore were removed before further analysis. The remaining sequences were realigned and phylogenetic analysis performed as described above. In this study, *Irx* gene numbering is adopted from Aase-Remedios et al. [54], being based on the inferred ancestral syntenic order of the genes in arthropods. Note, however, that different naming schemes have been used in other studies [140, 141].

For the NK and NK2 clusters, the initial approach did not yield a full gene complement. Since manual searches in the transcriptome had identified candidate sequences for some of the missing genes (e.g., NK7), we theorized that the e-value cutoff was too strict. We repeated the genome scan but lowered the e-value cutoff to 10^−10^. Since this increased the probability that non-NK/NK2 homeobox might be detected, we also included the *lab* homeoboxes of *D. melanogaster*, *T. castaneum*, and *S. maritima* as outgroups. The analysis otherwise proceeded in the same manner.

During analysis of the NK/NK2 gene tree, we noticed a long branch in the *Hlx* clade that only contained *P. litorale* sequences. Full-length sequences in that branch were annotated by EggNOG-mapper as *Dbx*, another homeobox gene of the ANTP class, prompting further investigation. We manually created a lightweight version of the NK/NK2 homeobox alignment by keeping 5–6 sequences per gene family and added the *Dbx* homeobox sequences for *D. melanogaster*, *T. castaneum*, *A. bruennichi*, and *C. sculpturatus* [54]. We re-aligned the remaining homeoboxes with mafft and calculated a neighbor joining tree without bootstrapping with Jalview.

More details can be found on the GitLab repository [45] in the 07-analysis/ folder. Please refer to notebooks nk_finder.ipynb; hro_finder.ipynb; sine_finder.ipynb; irx_finder.ipynb for the respective gene families/clusters.).

### Self-synteny analysis

Beyond the study of conserved homeobox gene clusters, self-synteny analysis was performed to globally screen the *P. litorale* genome for striking signatures of a WGD. This was inspired by the Oxford grid [58], a visualization technique often used to identify orthologous syntenic blocks between two species [142]. To this end, we performed an all-against-all search with the predicted peptide sequences of the gene models using mmseqs2 easy-search (–cov-mode 5 -c 0.8). We used the default mmseqs2 e-value threshold of 10^−4^, to detect the less obvious sequence similarity expected for paralogous sequences.

The alignment results were filtered to exclude self-hits and only include the best-scoring alignment for each gene pair; this was done to avoid counting matches caused by isoforms multiple times. Additionally, in cases where a gene had multiple putative paralogs on the same pseudochromosome, only the best hit (by e-value) was kept. This was done to prevent miscounting between two pseudochromosomes with multiple genes of the same family. At the lower e-value thresholds required to detect paralogs, all genes in one pseudochromosome would match all genes in the other, artificially inflating the strength of the connection between the two pseudochromosomes.

To create the Oxford grid, we plotted the chromosomes in linear, ascending order. Each point on the Oxford grid represents a pair of genes *g*_*i*_ and *g*_*j*_, where *g*_*j*_ was found using *g*_*i*_ as the query; this means that the Oxford grid is approximately but not entirely symmetric. The coordinate of a gene *g*_*i*_ that lies on pseudochromosome *k* with length *L*_*k*_ is calculated as follows:

$$\text{coord}\left({g}_{i}\right)={\sum }_{n=0}^{k-1}{L}_{n}+ \frac{(star{t}_{{g}_{i}}+en{d}_{{g}_{i}})}{2}$$

More details can be found in the GitLab repository [45] under 07-analysis/self_synteny.ipynb.

## Supplementary Information

Additional file 1. Fig. S1. Genome size estimation from k-mer (k = 21) coverage statistics. A) GenomeScope profile for PacBio reads; B) GenomeScope profile for ONT reads; C) GenomeScope2 profile for PacBio reads; D) GenomeScope2 profile for ONT reads. Fig. S2. Visualization of the best hits for chelicerate *abdA* sequences on pseudochromosome 56, in relation to the location of the *P. litorale Hox7/Antp* and *Hox8/Ubx* gene models. Notably, no hits are found between the gene models, where a presumptive *abdA* locus would be expected. For the accession IDs and sequences used for this, refer to Additional File 21, also available on Zenodo [145]. Fig. S3. Distribution of paralog content for different e-value thresholds. Gaussian kernel density estimates (covariance factor λ = 0.25) calculated from the histograms of putative paralog content for each pseudochromosome. More details can be found in the corresponding notebook (https://gitlab.phaidra.org/zoology/plit-genome under 07-analysis/self_synteny.ipynb). Fig. S4. Overview of A) (pseudo-)chromosome number and B) BUSCO completeness for different published arthropod genome assemblies. Each point represents one genome, with Hexapoda shown in yellow, chelicerates in magenta, myriapods in gray, and crustaceans in cyan. The bisected point shows the average of the distribution. The dashed red line denotes the values for *P. litorale* (this study). The underlying data can be found in Additional File 20: Table 11 [145]

Additional file 2. Table 1. Overview of the sequencing data generated for the project. Technology: sequencing platform used. De novo: whether the data was used to assemble a de-novo transcriptome. Annotation: whether the transcriptomic data was used to predict protein-coding genes. Accession: European Nucleotide Archive Accession IDs. Developmental staging following [37–39]. Table 2. QUAST and BUSCO Arthropoda scores that document the progress of the Flye-based assembly. Table 3. presence/absence overview of microRNA families predicted in the *Pycnogonum litorale* genome by MirMachine and supplemented with manual curation. Table 4. overview of gene models and the names assigned via phylogenetic analysis. Table 5. Overview of the best hits for chelicerate *abdA* sequences (also see Additional File 1: Fig. S4). The underlying sequences can be found in Additional File 8 (https://explore.openaire.eu/search/dataset?pid=10.5281%2Fzenodo.14362378). Gene tree for the *P. litorale* Hox cluster. *P. litorale* gene models are highlighted with stars and transcripts with triangles. Colors follow the scheme from [49]. Bootstrap support values are noted on the branches. Table 6: List of NCBI BLAST hits for gene model r2_g3735, predicted to reside in the Hox cluster of *P. litorale*. Gene tree for the *P. litorale* HRO cluster. *P. litorale* gene models are highlighted with blue squares. The paraphyletic Hbn tree has not been colored. Bootstrap support values are noted on the branches. Gene tree for the *P. litorale* IRX cluster. *P. litorale* gene models highlighted with blue squares. Bootstrap support values are noted on the branches. Gene tree for the *P. litorale* SINE cluster. *P. litorale* gene models highlighted with dark blue squares. Bootstrap support values are noted on the branches. Gene tree for the *P. litorale* NK/NK2 cluster. *P. litorale* gene models highlighted with dark blue squares. Colors follow the scheme from [49]. Bootstrap support values are noted on the branches. Reduced gene tree for the *P. litorale* NK/NK2 cluster including Dbx sequences. Numbers on the branches are distances as calculated in the neighbor-joining tree. Color is automatically applied by Jalview to visually separate clades at the chosen cut (red line). *P. litorale* gene models include g1744, g1756, g11364, and at_DN2391. Comparison of COX1 (cytochrome oxidase I) sequences between lab culture (labelled “Helgoland”), the wildtype animals used (labelled “Maine”) and the NCBI entries MG934985, MG935177, MG935394, and HM425354 (*P. litorale* COX1 partial CDS). Genome alignment produced by Geneious v10.2.6. Comparison of COX1 (cytochrome oxidase I) sequence identity between lab culture (labelled “Helgoland”), the wildtype animals used (labelled “Maine”) and the NCBI entries MG934985, MG935177, MG935394, and HM425354 (*P. litorale* COX1 partial CDS). Table 7. Iso-seq mixing strategy for the various developmental stages. We aimed for an approximately equimolar mix while trying to reach recommended concentrations for PacBio sequencing and considering the total amount of RNA extracted from each developmental stage. The RIN number is a score of RNA integrity [146] with values ranging from 10 (intact) to 1 (totally degraded); DV200 denotes the percentage of RNA fragments with length greater than 200 nucleotides. Table 8. Chelicerate repeat content, broken down by common repeat families. Manually extracted from various publications. Table 9: Table of genome assembly statistics for arthropod genomes. Obtained from NCBI. Table 10. Arthropod repeat content. Chelicerate, myriapod, and hexapod data as reported by Sheffer et al*.* in Table 4 [47]. Crustacean data as reported by Cui et al*.* [147]. Table 11. List of chelicerate reference genome assemblies found on NCBI that were at least scaffold level. Ticks and mites are overrepresented; when multiple species from the same genus were present, we chose the one with more genes, as generally most ticks and mites with sequenced genomes are parasitic and have reduced genomes. Among the remaining chelicerate taxa, spiders are overrepresented; here we chose the species with the least predicted gene models for each genus, in the hope that we would avoid false positives. We excluded *A. ventricosus* as it contains an uncharacteristic number of predicted proteins. The number of genes corresponds to the total number of genes in the annotation, not the protein-coding ones. Gene-level analyses. Includes folders for the analysis of the HOX, NK/NK2, HRO, IRX, and SINE clusters, as well as the r2_g3735 gene. The Hox subfolder also contains the sequences and alignments used for the abdA transcriptome search. More details can be found in the corresponding notebooks [45] (under 07-analysis/). Table 12: Mmseqs2 alignment of the predicted *P. litorale* proteome against itself. The columns hold, in order: query sequence identifier, target sequence identifier, fraction of identical matches (fident), alignment length (number of aligned columns), number of mismatches, number of gap open events, alignment start position in the query, alignment end position in the query, alignment start position in the target, alignment end position in the target, e-value of the alignment, bit score of the alignment, query sequence length. Underlies the self-synteny analysis.

Additional file 3.

## Data Availability

All relevant code for data preprocessing, genome and transcriptome assembly, subsequent analysis, and figure generation is available at GitLab under https://gitlab.phaidra.org/zoology/plit-genome. An archived version of the code can be found on Zenodo (https://zenodo.org/records/14920283 [143]). The computational results of this work have been achieved using the Life Science Compute Cluster (LiSC) of the University of Vienna. At the time of writing, LiSC is operating Oracle Linux Server v9.4 and uses SLURM [144] for the management of computational tasks. The code provided here is optimized for this environment but is easily adapted to any UNIX-based system. The computing environments for certain tools were modularized as conda environments. A list of configuration files for the most recent environment per tool can be found in the repository as well. The datasets supporting the conclusions of this article are available in the ENA repository (Study Accession PRJEB80537). An overview table can be found in Additional File 2: Table 1. Intermediate results, configuration files, and data required for figures are available as Additional Files or on Zenodo (https://explore.openaire.eu/search/dataset?pid=10.5281%2Fzenodo.14362378 [145]).

## Abbreviations

WGD: Whole-Genome duplication
ONT: Oxford Nanopore Technologies
PacBio: Pacific Biosciences
BUSCO: Benchmarking Universal Single-Copy Orthologs
HCR-FISH: Hybridization chain reaction fluorescent in-situ hybridization
TE: Transposable element
COX1: Cytochrome C oxidase subunit I
VBC: Vienna BioCenter
HMW: High molecular weight
PCR: Polymerase chain reaction
ORF: Open reading frame
