# Supplementary material for: The genome of a sea spider corroborates a shared Hox cluster motif in arthropods with a reduced posterior tagma
Source: BMC Biol. 2025 Jul 2;23:196. doi: 10.1186/s12915-025-02276-x (PMC12220506; doi:10.1186/s12915-025-02276-x)
Supplement: Supplementary file 1 — Additional file 1. Fig. S1. Genome size estimation from k-mer (k = 21) coverage statistics. A) GenomeScope profile for PacBio reads; B) GenomeScope profile for ONT reads; C) GenomeScope2 profile for PacBio reads; D) GenomeScope2 profile for ONT reads. Fig. S2. Visualization of the best hits for chelicerate abdA sequences on pseudochromosome 56, in relation to the location of the P. litorale Hox7/Antp and Hox8/Ubx gene models. Notably, no hits are found between the gene models, where a presumptive abdA locus would be expected. For the accession IDs and sequences used for this, refer to Additional File 21, also available on Zenodo [145]. Fig. S3. Distribution of paralog content for different e-value thresholds. Gaussian kernel density estimates (covariance factor λ = 0.25) calculated from the histograms of putative paralog content for each pseudochromosome. More details can be found in the corresponding notebook (https://gitlab.phaidra.org/zoology/plit-genome under 07-analysis/self_synteny.ipynb). Fig. S4. Overview of A) (pseudo-)chromosome number and B) BUSCO completeness for different published arthropod genome assemblies. Each point represents one genome, with Hexapoda shown in yellow, chelicerates in magenta, myriapods in gray, and crustaceans in cyan. The bisected point shows the average of the distribution. The dashed red line denotes the values for P. litorale (this study). The underlying data can be found in Additional File 20: Table 11 [145] [file 12915_2025_2276_MOESM1_ESM.pdf]

The genome of a sea spider corroborates a shared Hox cluster motif in arthropods with a reduced posterior tagma – Supplementary File 1

Papadopoulos, Nikolaos[1]*; Kulkarni, Siddharth S.[2,4]; Baranyi, Christian[1]; Fromm, Bastian[3]; Setton, Emily V.W.[4,5]; Sharma, Prashant P.[4]; Wanninger, Andreas[1]*; and Brenneis, Georg[1]*

[1] Integrative Zoology Unit, Department of Evolutionary Biology, University of Vienna, Vienna, Austria
[2] CSIR-Centre for Cellular and Molecular Biology, Hyderabad, India
[3] The Arctic University Museum of Norway, UiT - The Arctic University of Norway, Tromsø, Norway
[4] Department of Integrative Biology and Zoological Museum, University of Wisconsin-Madison, Madison, Wisconsin, USA
[5] The Whitney Laboratory for Marine Bioscience, Department of Biology, University of Florida, St. Augustine, Florida, USA
* to whom correspondence should be addressed

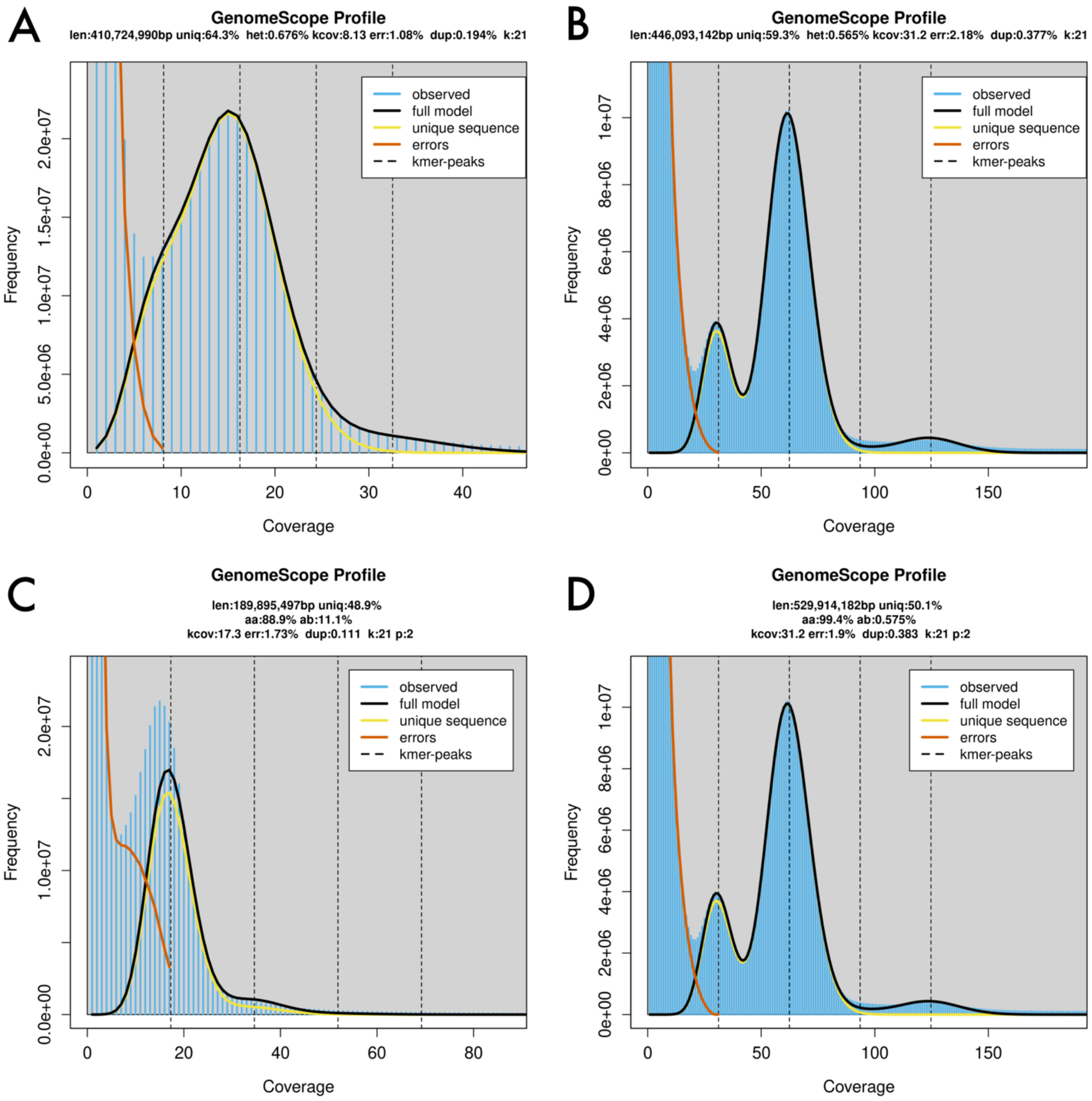

Fig. S1: Genome size estimation from k-mer (k=21) coverage statistics. A) GenomeScope profile for PacBio reads; B) GenomeScope profile for ONT reads; C) GenomeScope2 profile for PacBio reads; D) GenomeScope2 profile for ONT reads.

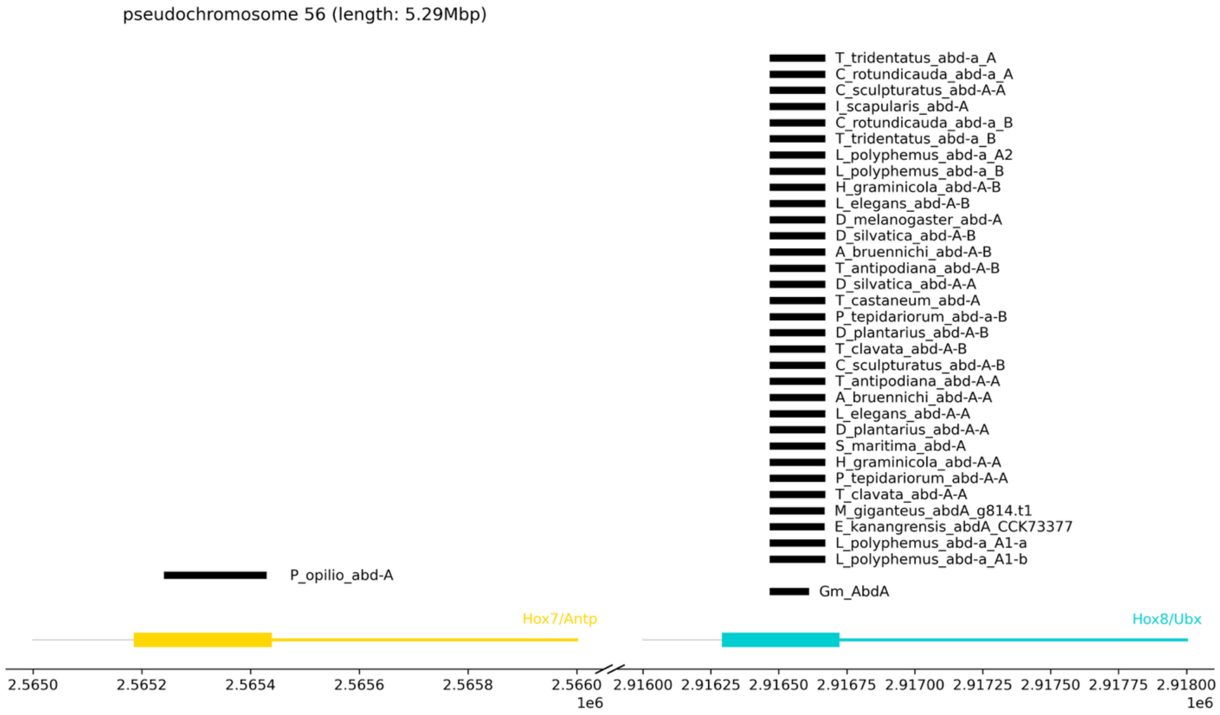

Fig. S2: Visualization of the best hits for chelicerate *abdA* sequences on pseudochromosome 56, in relation to the location of the *P. litorale Hox7/Antp* and *Hox8/Ubx* gene models. Notably, no hits are found between the gene models, where a presumptive *abdA* locus would be expected. For the accession IDs and sequences used for this, refer to Additional File 21, available on Zenodo (https://explore.openaire.eu/search/dataset?pid=10.5281%2Fzenodo.14362378).

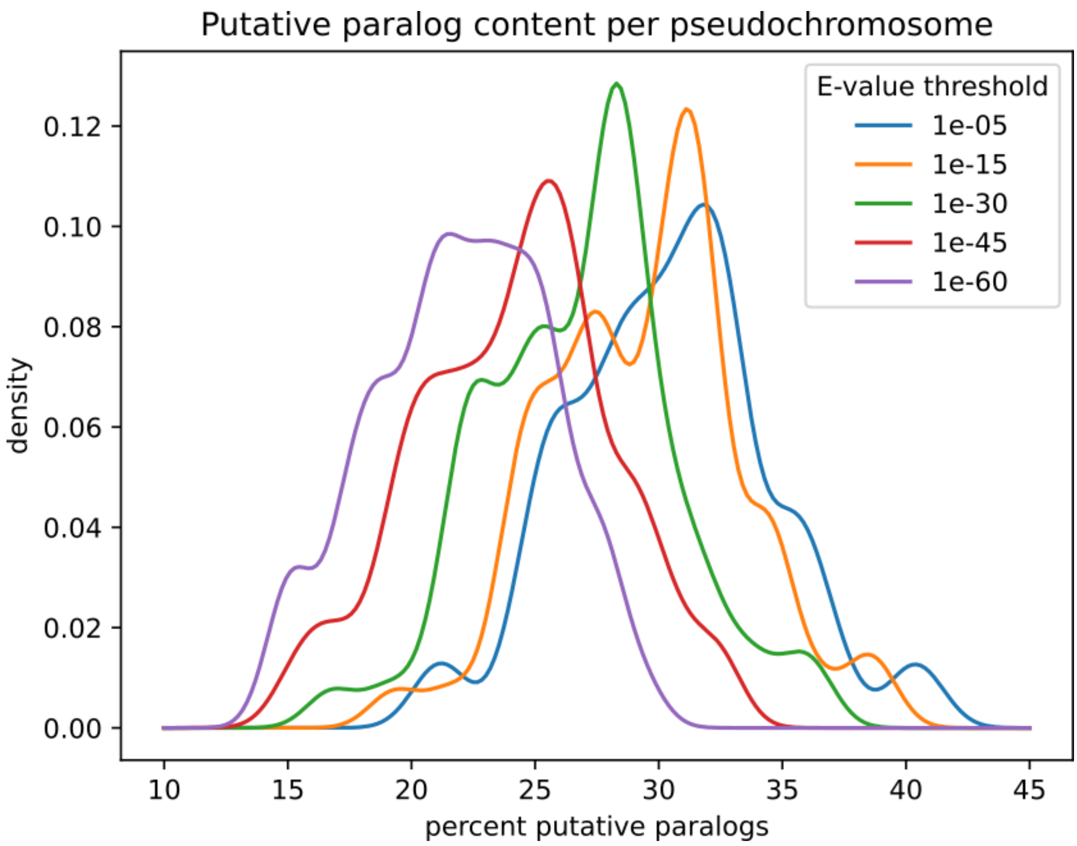

Fig. S3: Distribution of paralog content for different e-value thresholds. Gaussian kernel density estimates (covariance factor λ=0.25) calculated from the histograms of putative paralog content for each pseudochromosome. More details can be found in the corresponding notebook (https://gitlab.phaidra.org/zoology/plit-genome under 07-analysis/self_synteny.ipynb).

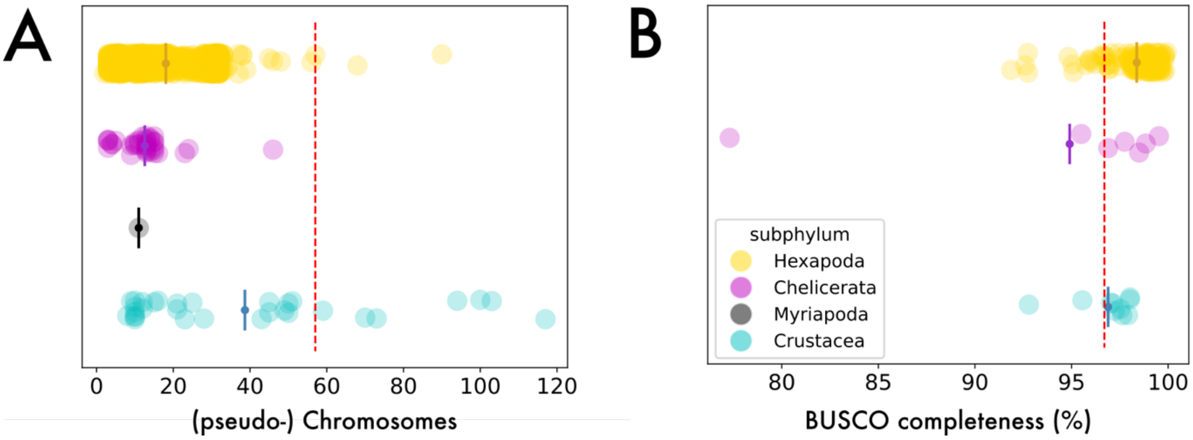

Fig. S4: Overview of A) (pseudo-)chromosome number and B) BUSCO completeness for different published arthropod genome assemblies. Each point represents one genome, with Hexapoda shown in yellow, chelicerates in magenta, myriapods in gray, and crustaceans in cyan. The bisected point shows the average of the distribution. The dashed red line denotes the values for *P. litorale* (this study). The underlying data can be found in Additional File 20: Table 11 (https://explore.openaire.eu/search/dataset?pid=10.5281%2Fzenodo.14362378).
