# Supplementary material for: The genome of a sea spider corroborates a shared Hox cluster motif in arthropods with a reduced posterior tagma
Source: BMC Biol. 2025 Jul 2;23:196. doi: 10.1186/s12915-025-02276-x (PMC12220506; doi:10.1186/s12915-025-02276-x)

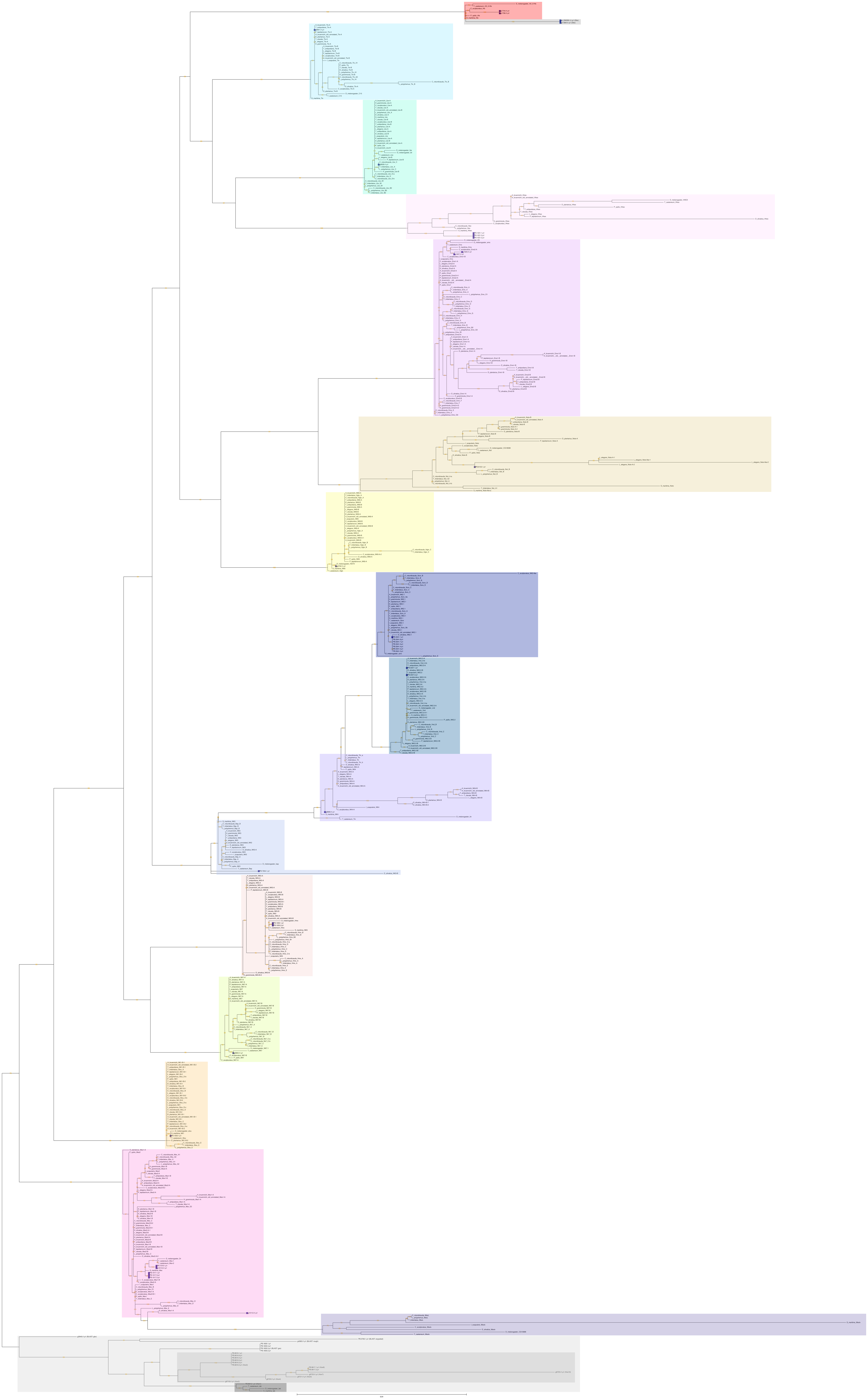

Supplement: Supplementary file 2 — Additional file 2. Table 1. Overview of the sequencing data generated for the project. Technology: sequencing platform used. De novo: whether the data was used to assemble a de-novo transcriptome. Annotation: whether the transcriptomic data was used to predict protein-coding genes. Accession: European Nucleotide Archive Accession IDs. Developmental staging following [37–39]. Table 2. QUAST and BUSCO Arthropoda scores that document the progress of the Flye-based assembly. Table 3. presence/absence overview of microRNA families predicted in the Pycnogonum litorale genome by MirMachine and supplemented with manual curation. Table 4. overview of gene models and the names assigned via phylogenetic analysis. Table 5. Overview of the best hits for chelicerate abdA sequences (also see Additional File 1: Fig. S4). The underlying sequences can be found in Additional File 8 (https://explore.openaire.eu/search/dataset?pid=10.5281%2Fzenodo.14362378). Gene tree for the P. litorale Hox cluster. P. litorale gene models are highlighted with stars and transcripts with triangles. Colors follow the scheme from [49]. Bootstrap support values are noted on the branches. Table 6: List of NCBI BLAST hits for gene model r2_g3735, predicted to reside in the Hox cluster of P. litorale. Gene tree for the P. litorale HRO cluster. P. litorale gene models are highlighted with blue squares. The paraphyletic Hbn tree has not been colored. Bootstrap support values are noted on the branches. Gene tree for the P. litorale IRX cluster. P. litorale gene models highlighted with blue squares. Bootstrap support values are noted on the branches. Gene tree for the P. litorale SINE cluster. P. litorale gene models highlighted with dark blue squares. Bootstrap support values are noted on the branches. Gene tree for the P. litorale NK/NK2 cluster. P. litorale gene models highlighted with dark blue squares. Colors follow the scheme from [49]. Bootstrap support values are noted on the branches. Reduced gene tree f [file add-file-12-nk_tree.pdf › add-file-12-nk_tree.pdf]
